# Circadian dynamics of the teleost skin immune-microbiome interface

**DOI:** 10.1186/s40168-021-01160-4

**Published:** 2021-11-16

**Authors:** Amy R. Ellison, David Wilcockson, Jo Cable

**Affiliations:** 1 School of Natural Sciences, Bangor University, Bangor, LL57 2DG UK; 2 Institute of Biological, Environmental and Rural Sciences (IBERS), Aberystwyth University, Aberystwyth, SY23 3DA UK; 3 School of Biosciences, Cardiff University, Cardiff, CF10 3AX UK

**Keywords:** Circadian rhythm, Clock gene expression, Microbiome, Parasite infection, Fish, Aquaculture, Photoperiod, Immunity, Metagenome

## Abstract

**Background:**

Circadian rhythms of host immune activity and their microbiomes are likely pivotal to health and disease resistance. The integration of chronotherapeutic approaches to disease mitigation in managed animals, however, is yet to be realised. In aquaculture, light manipulation is commonly used to enhance growth and control reproduction but may have unknown negative consequences for animal health. Infectious diseases are a major barrier to sustainable aquaculture and understanding the circadian dynamics of fish immunity and crosstalk with the microbiome is urgently needed.

**Results:**

Here, using rainbow trout (*Oncorhynchus mykiss*) as a model, we combine 16S rRNA metabarcoding, metagenomic sequencing and direct mRNA quantification methods to simultaneously characterise the circadian dynamics of skin clock and immune gene expression, and daily changes of skin microbiota. We demonstrate daily rhythms in fish skin immune expression and microbiomes, which are modulated by photoperiod and parasitic lice infection. We identify putative associations of host clock and immune gene profiles with microbial composition. Our results suggest circadian perturbation, that shifts the magnitude and timing of immune and microbiota activity, is detrimental to fish health.

**Conclusions:**

The substantial circadian dynamics and fish host expression-microbiome relationships we find represent a valuable foundation for investigating the utility of chronotherapies in aquaculture, and more broadly contributes to our understanding of the role of microbiomes in circadian health of vertebrates.

Video Abstract

**Supplementary Information:**

The online version contains supplementary material available at 10.1186/s40168-021-01160-4.

## Introduction

Circadian rhythms—endogenous daily cycles in physiological and behavioural processes—are a ubiquitous phenomenon to life. Living organisms are adapted to anticipate the daily variations in light, temperature, or food availability driven by the relentless 24-h rotation of Earth. Circadian rhythms are orchestrated by ‘clock genes’ driving transcriptional-translational autoregulatory feedback loops [1], which are transduced to temporally coordinate biological activities. Immune functions are energetically costly [2] and often highly rhythmic, enabling organisms to mount their most efficient response at times when risk of infection or injury is highest [3–5]. Conversely, immune factors and infections can affect expression of molecular clocks [6–8] and subsequent rhythmic phenotypes [9, 10]. Disruption of normal circadian cycles can impact immune functioning [11, 12] and may increase disease risks [13].

A primary function of immune systems is to protect the host from invading pathogenic microbes. However, animals are invariably colonised by a suite of microorganisms—their ‘microbiome’—which span the spectrum of symbiosis from mutualists to opportunistic pathogens. In vertebrates, it is increasingly apparent that immune systems and microbiomes are intricately linked, together mediating homeostasis and influencing disease outcomes [14, 15]. Intriguingly, microbiomes may also be rhythmic, exhibiting diurnal fluctuations in community composition and activity [16]. In studies of the mammalian gut, it has been demonstrated that not only does expression of host clock genes shape microbiome rhythms [17] but also disruption of microbial rhythms in turn impacts host circadian functioning [18].

Aquaculture is the world’s fastest growing food sector, but infectious disease is the principle barrier to sustainability [19] and a multi-billion-dollar problem for the global industry [20]. Whilst understanding of fish microbiomes is still in its infancy compared to mammalian systems, there is rapidly growing interest in their role for fish nutrition, health and disease resistance [21–25]. Photoperiod manipulation is commonly used in fish farms, with extended day lengths, and, in the extreme, constant light, to promote increased growth rates, or control maturation and reproduction [26–28]. Fish are thought to have a decentralised clock, with cells from multiple tissues expressing circadian genes [29, 30], self-sustained rhythmicity and light responsiveness (see [31] for review). In common with higher vertebrates, fish appear to exhibit circadian rhythmicity in certain immune factors [29, 30, 32–35]. Therefore, extreme lighting regimes may have profound implications for fish health and response to infection. Moreover, there are indications that infection and/or stress may impact expression of fish circadian clocks [36, 37]. Currently, the extent to which light manipulation practices contribute to disease in aquaculture is unknown. More fundamentally, the daily dynamics of the fish immune-microbiome interface is yet to be explored. Uncovering the effects of infection and photoperiod on fish immune and microbiome rhythms will be pivotal for both aquaculture disease mitigation strategies, and a broader understanding of the role of holobiont chronobiological interactions for animal health.

Here, using rainbow trout (*Oncorhynchus mykiss*) as a model, we combine 16S rRNA gene metabarcoding and direct mRNA quantification methods to simultaneously characterise the circadian dynamics of skin clock and immune gene expression, and daily changes of skin microbiota. We compare circadian rhythms of host clock and immune gene expression and microbial community composition in healthy fish under regular light-dark cycles (12 h light, 12 h dark, hereafter 12:12 LD) with those in fish experimentally infected with the ectoparasite crustacean *Argulus foliaceus* and/or raised under constant light (24:0 LD). In addition, we assess rhythmicity in the functional potential of rainbow trout skin microbiomes and establish host expression-microbiome association networks.

## Results

### Photoperiod impacts host responses to infection

**Fig. 1:**
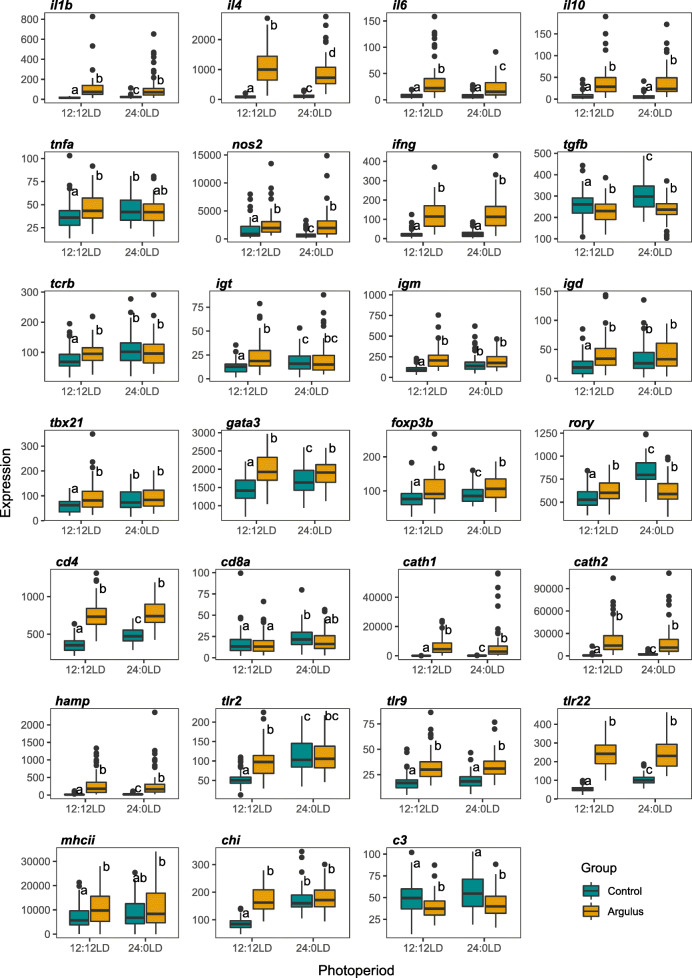
Expression of immune genes in uninfected (control; cyan) and *Argulus*-infected (orange) rainbow trout maintained under 12:12 LD and 24:0 LD conditions. Letters denote significant differences in expression between groups. Expression is normalised counts of mRNA copies detected via Nanostring nCounter

Photoperiod (12:12 LD vs 24:0 LD) had no significant impact on growth of juvenile rainbow trout over the 16-week trial period (Supplementary Figures 1a and 1b). However, a significantly higher number of *Argulus* lice survived 7 days post-inoculation on fish maintained in constant light conditions (*t*_115_ = −8.418, *P* = 1.23 × 10^−23^, Supplementary Figure 1c). To examine overall immune responses to *Argulus* infection, we grouped fish from all timepoints, and contrasted expression of 27 genes from innate and adaptive immune pathways between treatment groups (12:12 LD control, 12:12 LD infected, 24:0 LD control, 24:0 LD infected). Infected rainbow trout had significantly higher expression of 24 immune genes (89%) under 12:12 LD, whereas only 14 (52%) were significantly higher in infected fish compared to healthy controls under constant light (Fig. 1). Two genes (*c3* and *tgfb*) were significantly reduced by infection in both light conditions (Fig. 1). Expression levels were broadly similar amongst infected groups, although upregulation of the pro-inflammatory interleukins *il4* and *il6* was lower under constant light (Fig. 1). Conversely, comparisons of healthy (unchallenged) fish under 12:12 LD and 24:0 LD revealed a substantial difference in immune expression profiles, with unchallenged fish under constant light exhibiting elevated expression levels in 21 genes (78%), more similar to both infected groups in most immune genes (Fig. 1).

### Circadian rhythmicity of host expression is altered by infection and photoperiod

**Fig. 2:**
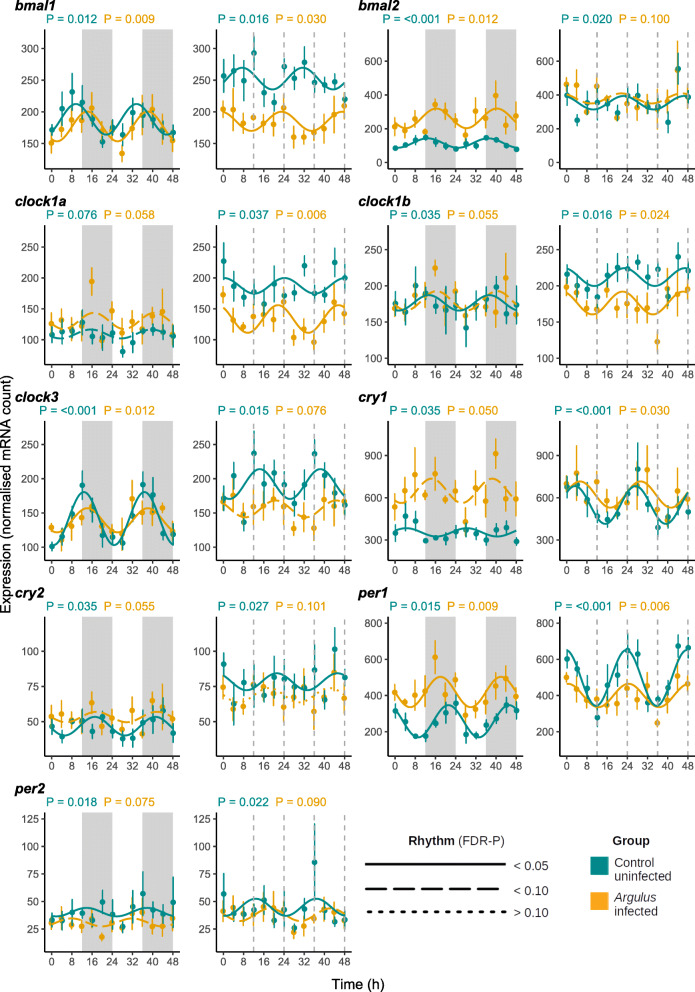
Mean expression (± 1 S.E.) of core clock genes of uninfected (cyan) and *Argulus*-infected (orange) rainbow trout maintained at 12:12 LD (left) and 24:0 LD (right). Expression is normalised counts of mRNA copies detected via Nanostring nCounter. Curves denote cosinor waveform fitted using CircaCompare. Grey shading indicates time periods in darkness (grey dashing indicates equivalent 12:12 LD light transitions on 24:0 LD plots)

Under 12:12 LD, core and accessory vertebrate clock genes exhibited significant circadian rhythmicity in healthy rainbow trout skin (Fig. 2, Supplementary Table 1, Supplementary Figure 2). Many of these genes are also found to be expressed rhythmically in fish raised in constant light (Fig. 2, Supplementary Table 1, Supplementary Figure 2) and when fish are placed into ‘free-running’ (constant dark, DD) conditions (Supplementary Figure 3, Supplementary Table 1). However, overall expression levels of clock genes are elevated in the absence of light cues (Fig. 2, Supplementary Figure 2), except for *timeless* (suppressed expression in 24:0 LD). In addition, *bmal2*, *clock1b*, *per1*, and *rora* exhibited a significantly different phase of expression in constant light (Supplementary Table 1, Fig. 2, Supplementary Figure 2).

*Argulus* lice infections had variable impacts on the expression levels and rhythmicity of the clock genes. When contrasted with healthy control groups, some gene rhythms were dampened in infected fish (i.e., significantly reduced amplitude; 12:12 LD *clock3*, 24:0 LD *per1*), rendered arrhythmic (*cry2* in 24:0 LD) and/or phase-shifted (*bmal1* in both light treatments, *cry1* and *per1* in 12:12 LD, *clock3* in 24:0 LD). Rhythms of clock gene expression in infected fish under the two photoperiod treatments did not differ in amplitude. But, *bmal1*, *clock1b*, *clock3*, *cry1*, *per1*, *per2*, *rory*, and *timeless* had significantly different phases of expression between infected fish under 12:12 LD and those raised in constant light. In addition, *bmal2*, *clock3*, *csnk1d*, *per2* and *reverbb* had increased rhythm mesors (average expression) in 24:0 LD, whilst *timeless* was suppressed (Supplementary Table 1, Fig. 2, Supplementary Figure 2).

Significant rhythmicity in expression was found in both innate and adaptive immune markers (Supplementary Table 1, Supplementary Figures 4 and 5), with a substantial proportion remaining rhythmic under free-running (DD) conditions (Supplementary Figure 6). The cathelicidins (*cath1*, *cath2*), *igd*, *il17a*, and *tbx21*, whilst rhythmic in healthy fish under 12:12 LD, were arrhythmic in fish maintained in constant light (Supplementary Table 1, Supplementary Figures 4 and 5). Of the immune genes rhythmic in healthy fish under both light conditions, the innate markers *chi*, *hamp* and *nos2*, and the adaptive markers *cd4*, *cd8a*, *foxp3b*, *igm*, *igt*, *tcrb* and *tgfb* had significantly different mesors; with the exception of *nos2*, all were more highly expressed in 24:0 LD. However, some of these more highly expressed genes (*cd4*, *foxp3b*, *hamp*, *igt*, *tgfb*), and others with similar expression levels between photoperiods (*il4*, *tlr9*), were phase-shifted in constant light (Supplementary Table 1, Supplementary Figures 4 and 5).

Fewer immune genes were rhythmically expressed in infected fish: 76% and 67% of rhythmic genes found in healthy fish were also rhythmic in the 12:12 LD and 24:0 LD infected groups respectively. Under 12:12 LD, the vast majority (94%) of the immune genes assayed with rhythmicity in both healthy and infected fish exhibited higher mesors in the infected group. In contrast, only 57% of immune genes with rhythms in healthy and infected fish in 24:0 LD had different expression levels (Supplementary Table 1). Only *tbx21* had a significantly altered amplitude in rhythm, with a higher amplitude in infected fish at 12:12 LD compared to both healthy 12:12 LD fish and infected fish in constant light. *Argulus* infection also shifted the phase of expression of *mhcii* under 12:12 LD and *c3*, *nos2* and *igt* in 24:0 LD (Supplementary Table 1).

### Argulus infection impacts skin mucus microbiome communities

After read pre-processing, error correction, chimaera removal and filtering, a total of 1037 amplified sequence variants (ASVs) were found across all samples. Rarefaction curves confirmed a minimum read depth of 2000 was sufficient to reach saturation of diversity in rainbow trout skin (Supplementary Figure 7a). Background water samples were distinct from fish groups (Supplementary Figure 7b) and had a significantly higher alpha diversity (average Faith’s phylogenetic distance: Control 12:12 LD = 7.10, Infected 12:12 LD = 7.63, Control 24:0 LD = 5.59, Infected 24:0 LD = 7.63, Water = 20.67, Supplementary Table 2, Supplementary Figure 7c). Contrasts of alpha diversity amongst fish samples revealed that the microbiomes of healthy fish under constant light were significantly less diverse than all other groups (all pairwise Kruskal-Wallis tests *P* < 0.001, Supplementary Table 2). Multivariate permutational analysis of beta diversity indicated significant compositional differences amongst all groups (Supplementary Figure 7b, Supplementary Table 3). Metagenomic sequencing of a subset of samples resulted in six high quality (completion > 90%, contamination < 5%) and five moderate quality (> 60% completion, < 5% contamination) metagenomic assembled genomes (MAGs, Table 1).

**Table 1:** Summary of metagenomic assembled genomes (MAGs) in rainbow trout skin

Bin ID	Amphora2 phylotype	Size (Mbp)	No. of contigs	No. of genes	CheckM completion (%)	CheckM contamination (%)
bin.14	Unknown *Rhizobiales* sp. 1	4.36	220	349	99.13	1.37
bin.16	Unknown *Oxalobacteraceae* sp. 1	6.34	83	574	95.52	1.00
bin.15	*Variovorax paradoxus 1*	6.23	236	427	93.84	2.58
bin.20	Unknown *Oxalobacteraceae* sp. 2	5.45	683	574	92.89	1.42
bin.12	*Microbacterium testaceum 1*	3.4	100	400	92.22	1.77
bin.17	*Microbacterium testaceum* 2	3.51	85	400	90.88	2.98
bin.11	*Pseudomonas* sp.	4.39	31	813	84.74	1.19
bin.4	*Variovorax paradoxus* 2	6.19	287	427	82.69	0.71
bin.19	Unknown *Moraxellaceae* sp.	1.74	338	451	70.41	2.05
bin.10	Unknown *Rhizobiales* sp. 2	2.48	556	408	64.70	0.82
bin.3	*Flavobacterium* sp.	2.29	482	511	61.54	0.79

**Fig. 3:**
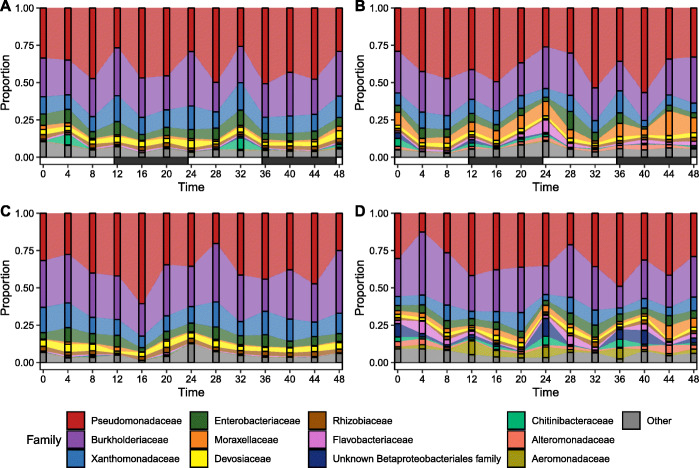
Alluvial plots of most abundant bacteria families (average > 1% across all data) in healthy (**A**, **C**) and *Argulus foliaceus* infected (**B**, **D**) trout under 12:12 LD (**A**, **B**) and 24:0 LD (**C**, **D**) photoperiods. Horizontal bars indicate periods of light (white) and dark (black)

**Fig. 4:**
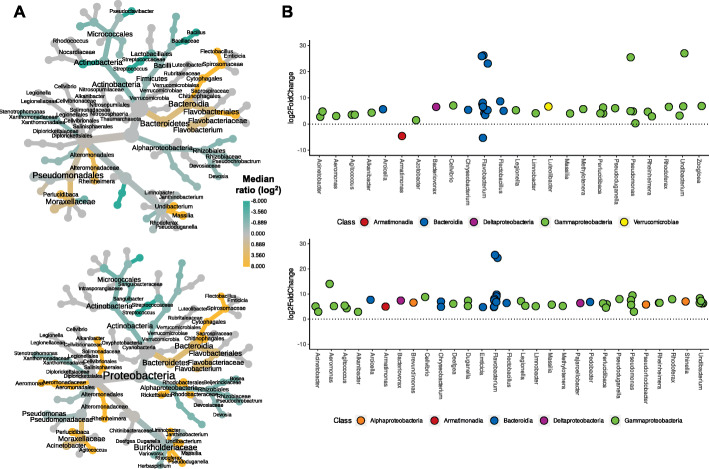
**A** Heat trees contrasting bacteria taxa abundance between healthy and *Argulus foliaceus* infected fish under 12:12 LD (top) or 24:0 LD (bottom) photoperiods. The colour of each taxon represents the log-2 ratio of median proportions of reads. Taxa with significant differences are labelled, determined using a Wilcox rank-sum test followed by a Benjamini-Hochberg (FDR) correction for multiple comparisons. Taxa coloured cyan are enriched in healthy fish and those coloured orange are enriched in infected fish. Node size is relative to prevalence in all samples. **B** Taxa with significantly different abundances (FDR-corrected *p* value < 0.05) between healthy and *A. foliaceus* infected fish under 12:12 LD (top) or 24:0 LD (bottom) photoperiods, determined via DESeq2 analyses. Taxa above the dotted line are significantly more abundant in infected fish; below the line are more abundant in healthy fish

The skin microbiome communities—determined by 16S rRNA profiling—in all groups were dominated by *Proteobacteria*, with Pseudomonadaceae and Burkholderiaceae accounting for over 50% of the communities in all groups and timepoints (Fig. 3). Taxonomic assignment of metagenomic reads revealed similar profiles, including dominance of the genus *Pseudomonas* (Supplementary Figure 8). The most abundant MAG was determined to be a *Pseudomonas* species from the *P. fluorescens* lineage [38], most closely related to *P. chloraphis* (Supplementary Figures 9 and 10). Wilcoxon rank-sum testing and DESeq2 on 16S data both revealed substantial differences in the relative abundances of microbial taxa between healthy and lice-infected fish (Fig. 4). At the higher taxonomic levels, healthy fish under both light treatments had a greater proportion of *Actinobacteria* and *Firmicutes* lineages, whilst both infected fish groups had increased *Bacterodia* lineages (Fig. 4A). At the genus level, many *Gammaproteobacteria* were more abundant in both infected groups (e.g. *Aeromonas*, *Perlucidibaca*, *Undibacterium*, Fig. 4B). *Bacteroidia* genera, including several *Chryseobacterium*, *Flectobacillus* and *Flavobacterium* ASVs, were also increased in infected fish, with *Flavobacterium* accounting for some of the highest fold-changes in abundance (Fig. 4B). One MAG was phylotyped as *Flavobacterium* sp. (most closely related to the fish pathogen *F. columnare*, Supplementary Figure 11), which was significantly more abundant in infected fish (Supplementary Figure 9, Supplementary Table 4). Full lists of 16S rRNA differentially abundant taxa are provided in Supplementary Table 5.

16S rRNA functional prediction (using inferences based on reference genomes similarity [39]) of microbiomes revealed putative differences in the activity of microbial communities amongst healthy and infected fish. LefSe analyses indicated pathways enriched in healthy fish groups were predominantly degradative classes including amino acid, aromatic compound and carbohydrate degradation (Supplementary Table 6). In contrast, functional enrichment of lice-infected fish microbiomes was dominated by biosynthetic pathways in both light conditions, particularly those involved in cofactor, carrier and vitamin biosynthesis (Supplementary Table 6). Similarly, functional analyses of metagenomic data revealed substantial differences between healthy and infected fish (Supplementary Table 7). Like the 16S rRNA results, we found increased abundance of functions related to lipid, nucleotide and thiamine processing in infected fish, and increased degradation-related (e.g. urea cycle) and energy generation (e.g. fermentation) functions in healthy fish. Overall, in 16S rRNA functional predictions, a greater number of pathways were identified as differentially abundant between healthy and infected fish in 24:0 LD, suggestive of a greater disruption in microbiota functional potential due to parasitic infection in fish maintained under constant light.

### Circadian rhythmicity of skin microbiota and association with host gene expression

**Fig. 5:**
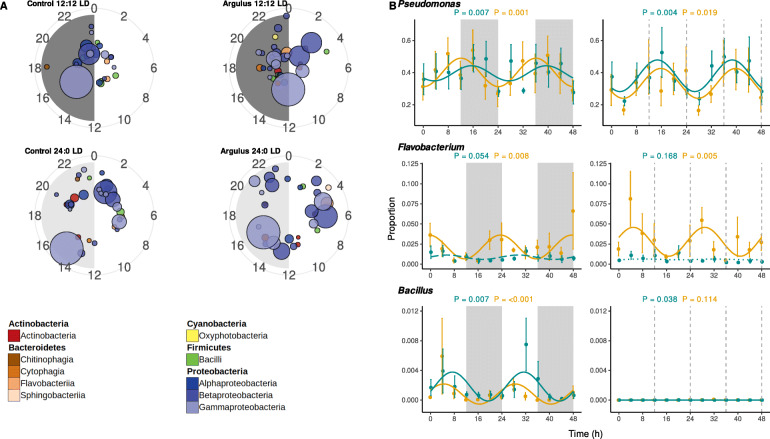
**A** Polar plots showing times of peak relative abundance of significantly rhythmic microbiome genera. Each circle represents a genus, coloured by class and scaled by average relative abundance. Radian indicates time of peak and distance from centre indicates significance (more significant/stronger rhythms towards edge of plot). **B** Examples of rhythmic bacteria genera (full results presented in Supplementary Table 8). Mean proportion of community (± 1 S.E.) of bacteria genera of uninfected (cyan) and *Argulus*-infected (orange) rainbow trout maintained at 12:12 LD (left) and 24:0 LD (right). Curves denote cosinor waveform fitted using CircaCompare. Grey shading indicates time periods in darkness (grey dashing indicates equivalent 12:12 LD light transitions on 24:0 LD plots)

Circadian rhythmicity in 16S rRNA relative abundance was apparent in 49 skin bacteria genera in one or more of the treatment groups (Supplementary Table 8, Fig. 5). We also found significant differences in abundance of several MAGs between the two circadian timepoints used for full metagenomic analyses (Supplementary Figure 9, Supplementary Table 4), supporting profound daily shifts in community composition.

Of the 41 genera rhythmic in both healthy and infected fish at 12:12 LD, 17 (41.5%) had significantly different mesors. In contrast, 23 (60.5%) had significantly different mesors when comparing healthy and infected fish under constant light. *Perlucidibaca*, *Undibacterium*, and *Rhodoferax* had significantly greater rhythm amplitudes in infected fish under both light treatments. In addition, *Flectobacillus*, *Alkanibacter* and an unassigned *Burkholderiaceae* genus had higher rhythm amplitudes in infected 12:12 LD fish, whilst *Duganella* had higher amplitude in 24:0 LD infected fish only. Under 12:12 LD, lice infection significantly altered rhythm phases of seven bacteria genera (unknown *Rhizobiaceae*, unknown *Rickettsiales*, *Deefgea*, *Massilia*, unknown *Neisseriaceae*, unknown *Chitinophagales* and *Legionella*). *Pseudoclavibacter* was the only genus found to have altered rhythm phase in 24:0 LD healthy vs infected comparisons.

**Fig. 6:**
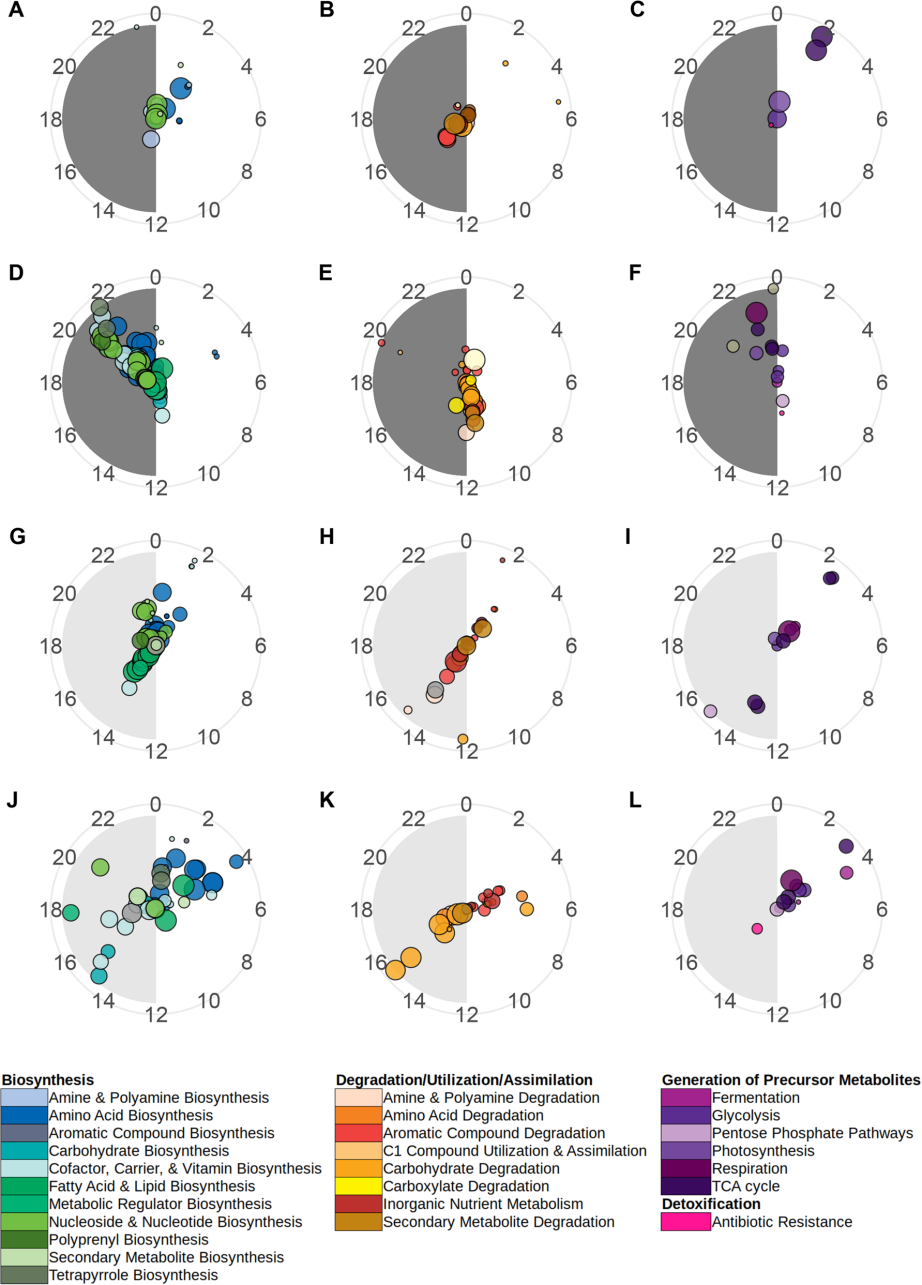
Polar plots showing peak relative abundance of significantly rhythmic microbiome MetaCycle pathways. Each circle represents a pathway, coloured by MetaCycle class and sized by average relative abundance. Pathway radian indicates time of peak and distance from centre indicates significance (more significant/stronger rhythms towards edge of plot). Pathway identity determined via Picrust2 and rhythmicity significance determined via eJTK_cycle (Bonferoni-corrected *P* values < 0.05). Circacompare was used to fit waveforms and determine estimates of rhythms peaks. **A**, **B**, **C** = healthy trout under 12:12 LD. **D**, **E**, **F** = *Argulus*-infected trout under 12:12 LD. **H**, **I**, **J** = healthy trout under 24:0 LD. **K**, **L**, **M** = *Argulus*-infected trout under 24:0 LD. Full details of pathways are provided in Supplementary Datafile 1

Visualisation of the timings of peak abundances of rhythmic taxa indicated no clear phylogenetic patterns (e.g. rhythmic *Proteobacteria* genera peak abundances were spread across the circadian cycle, Fig. 5A). However, when considering the rhythms of the functional potential of the microbiome communities, we found evidence of temporal patterns (Fig. 6). In healthy fish under 12:12 LD, the majority of rhythmic biosynthetic (e.g. heme b, L-lysine and isoprene biosynthesis) and energy generation (e.g. glycolysis, TCA cycle) functions peaked in the first hours of light, whilst degradation function peaks were found primarily in dark hours. In contrast, in infected fish under 12:12 LD, rhythmic biosynthetic and energy generation functions predominantly peak in abundance towards the end of the dark period, whilst degradation pathways peaked just before dark. Supporting these findings, analysis of metagenomic sequences revealed substantial differences in abundance of bacterial functions between the onset of light and dark periods in both healthy and infected fish (Supplementary Table 7).

Constant light conditions also appeared to shift the broad temporal patterns of function abundances. In healthy fish under 24:0 LD, many biosynthetic pathways (e.g. l-valine, heme b and enterobactin biosynthesis) peaked at a circadian time similar to those in the 12:12 LD group. However, we also found a large cluster of biosynthetic pathways peaking later in the day (e.g. fatty acid, spirillozanthin and coenzyme M biosynthesis). In infected fish under 24:0 LD, biosynthetic pathway rhythms were more dispersed, with peaks spread around the majority of the 24-h cycle. For degradation and generation of energy pathways in both healthy and infected fish under 24:0 LD, we found multiple clusters of peak abundances around the 24 h cycle, rather than a single predominant cluster as in 12:12 LD conditions (Fig. 6).

**Fig. 7:**
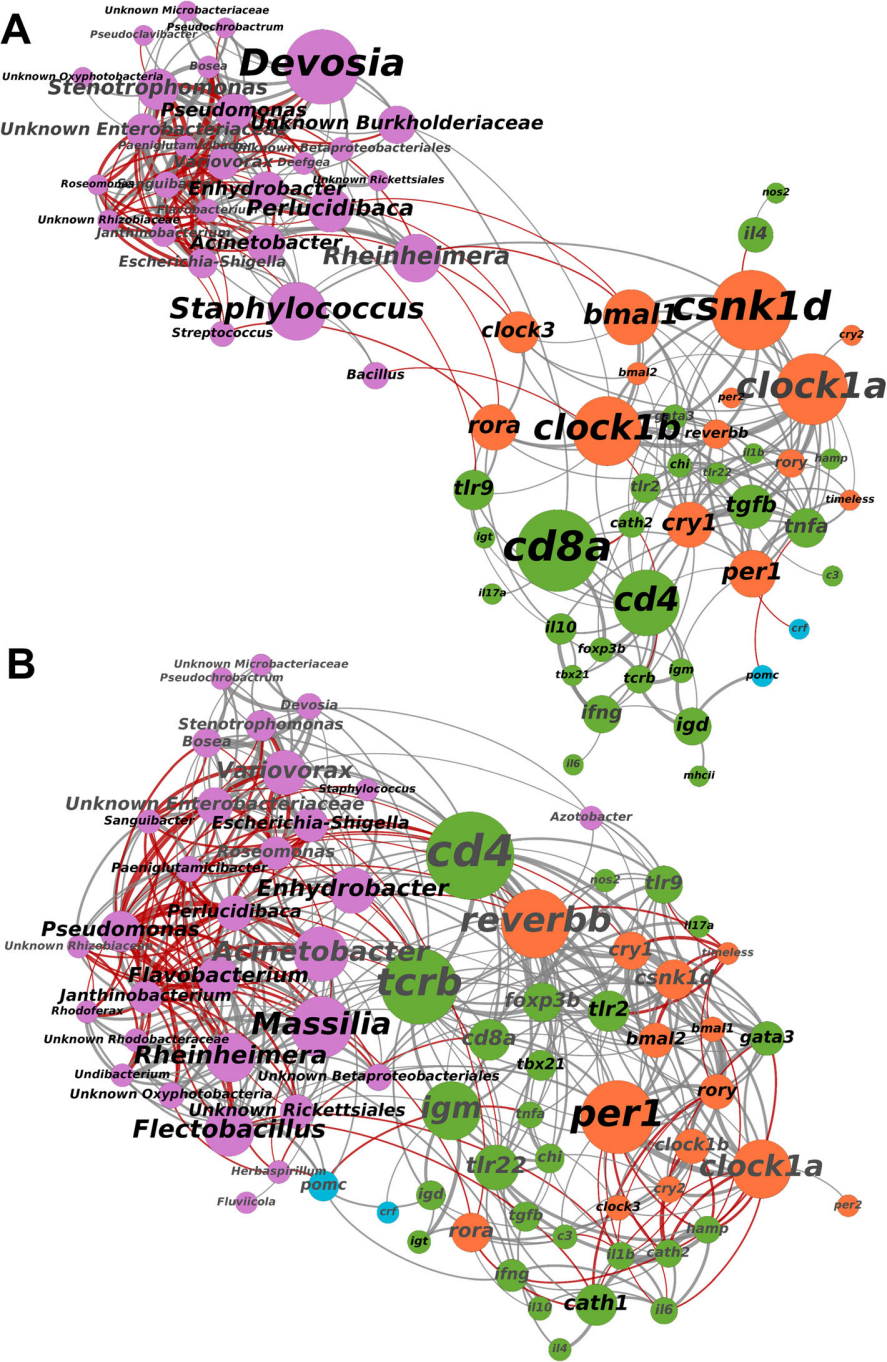
Co-occurrence networks of microbial genera (pink) and host gene expression (orange = clock, green = immune, blue = corticotropin) in healthy (panel **A**) and *Argulus*-infected (panel **B**) trout under 12:12 LD. Node and label size scaled to degree centrality score. Label colour denotes rhythmicity (black = rhythm FDR *p* value < 0.05, grey = rhythm FDR *p* value > 0.05). Connection colour indicates association (grey = positive, red = negative, determined by Spearman correlation tests) and connection width scaled to correlation strength (thicker lines denote a higher correlation coefficient)

We used co-occurrence network analyses to assess associations of host gene expression and their microbiomes, using betweeness centrality scores and number of connections (degrees) to identify influential genes and bacteria genera [40, 41]. In healthy 12:12 LD fish, there was a high level of connectivity within host immune and clock genes, and within microbial taxa (Fig. 7). Links across the gene expression and bacteria subnetworks were primarily via the rhythmically expressed clock genes *clock1b*, *clock3*, *bmal1*, *rora* and *csnk1d*. However, expression of the toll-like receptors *tlr2* and *tlr9* were significantly associated with abundance of *Bacillus* and *Enhydrobacter*, respectively. In contrast, networks of infected fish under 12:12 LD revealed a higher level of connectivity between host expression and bacteria (Fig. 7). The immune markers *cd4* and *tcrb*, and the clock gene *reverbb* were most influential in terms of their betweeness centrality scores and number of significantly associated microbial taxa (Fig. 7).

In contrast to 12:12 LD, clock genes were less influential (in terms of centrality) in gene-microbe networks for uninfected fish under constant light (Supplementary Figure 12). However, several immune genes (*igd*, *ifng*, *nos2*, *hamp*, *tcrb*, *foxp3b*) were significantly associated with one or more bacteria genera. *Tcrb* was most influential by betweeness centrality (expression positively correlated with *Janthinobacterium* and negatively with *Flavobacterium*), whilst *ifng* was linked to the highest number of taxa (*Escherichia*-*Shigella*, *Pseudomonas*, *Varioivorax*, *Stenotrophomonas* and *Pseudoclavibacter*). Similar to 12:12 LD contrasts, the network of infected fish under 24:0 LD showed a higher level of connectivity between host gene expression and microbiota compared to the healthy network (Supplementary Figure 12), with the immune markers *cd8a* and *tcrb* found to be the most influential genes (in terms of number of associations with taxa and centrality score).

## Discussion

We demonstrate the daily dynamics of immune expression and microbiome composition in fish skin and show ectoparasite infection and constant light—a commonly used environmental condition in aquaculture—can significantly alter circadian rhythms of immunity and microbiota, which may be detrimental for host disease resistance. In addition, network analyses of host gene expression and their microbiomes reveal clock expression, and T cell populations are likely key in shaping the skin host-microbiome interface of teleosts. Our examination of the skin circadian immune response to infection under extreme photic regimes are directly relevant to fish culture practices; fish peripheral tissues are thought to have entrainable, light-responsive clocks [31], which may make them particularly susceptible to negative health consequences from constant lighting as used in aquaculture.

Over our trial period, we found no significant difference in the growth of rainbow trout fry maintained under 12:12 LD and constant light (24:0 LD) when fish were provided equivalent food rations. However, when challenged with *Argulus* lice, their ability to clear infection was significantly altered by photoperiod. Under constant light, rainbow trout had a significantly higher lice burden 1 week after inoculation, indicating a reduced ability to mount an effective immune response. These findings are consistent with previous studies showing extended day length increases ectoparasite susceptibility and altered expression in specific immune genes in sticklebacks [42]. Immune profiles in uninfected fish showed elevated levels of expression in both innate and adaptive pathways under constant light. When infected with lice, rainbow trout under both photoperiods showed similar patterns of immune gene responses, except for the interleukins *il4* (mediator of Th2 differentiation) and *il6* (key to initiate inflammation) which were expressed at lower levels in constant light. Early inflammatory responses and subsequent initiation of Th2 processes are thought to be critical to resistance of crustacean ectoparasites in salmonids [43]. Taken together, chronic elevation of the immune gene expression—which may result in immune exhaustion [44] or other immunopathologies [45]—and reduced ability to mount effective responses key to lice resistance suggest rearing of fish in the absence of light cues are likely to be detrimental for health.

The impact of photoperiod on overall magnitude of immune gene activation is not the only factor important to parasite resistance; the rhythmicity and the appropriate timing of immune activity (i.e. when fish are maximally vulnerable to pathogen attack) may also be key to pathogen defences. Under regular light-dark cycles, we show rainbow trout skin is highly rhythmic in expression of the core vertebrate clock genes and many immune genes in both innate and adaptive pathways. In essence, we find the highest expression of pro-inflammatory markers (e.g. *il6*, *il17a*) at the onset of the light period and peaks in anti-microbial peptide genes (e.g. cathelecidins) mid-light phase, whilst immunoglobulin and T cell markers were highest during dark hours. The timing of different facets of immune systems are considered to have evolved to offer hosts greatest protection from invading pathogens when most likely to encounter them, whilst avoiding energetically inefficient and potentially immunopathological risk of continual immune activation [46]. We found that constant light resulted in arrhythmic expression of genes involved in mucosa anti-microbial (e.g. cathelecidins, *igd*, *il17a*) and Th1 (*tbx21*) responses. Furthermore, genes with phase-shifted expression rhythms in constant light were dominated by those involved in T cell differentiation and regulation (e.g. *cd4*, *foxp3b*, *il4*, *tgfb*). Loss of synchrony between host immunity and parasite activity and/or immune evasion rhythms is very likely to be detrimental for host fitness and survival [47]. Our results indicate that this is a factor in the reduced clearance of lice in fish reared in constant light. Clearly, the impacts of light cycle perturbation, be it intentional such as in aquaculture or unintentionally due to light pollution [48], must be more carefully considered for animal health.

The primary function of fish skin mucus is as a protective barrier and it hosts diverse communities of microbes [49] which are thought to contribute to protection from microbial pathogens via competitive and/or antagonistic activities [50, 51]. Whilst pathogenic taxa occur mostly at low levels in healthy teleost microbiomes, their proliferation is a common signal of microbiome perturbation and dysbiosis [52]. *Argulus* lice infestations are commonly observed alongside bacterial, fungal or viral infections [53]. Here, using evidence from both 16S rRNA profiling and metagenomic sequencing, we demonstrate significant reorganisation of bacterial communities and their potential functional activities in rainbow trout skin when infected with *A. foliaceus*, including notable increases in abundance of genera associated with infectious disease [54, 55]. *Flavobacterium* spp. were found to have marked increases in infected fish, and the *Flavobacterium* MAG (closely related to *F. columnare*, the agent of columnaris disease) was more abundant in *Argulus*-infected samples. Fish lice may elicit host immune profiles and/or destabilise skin microbiota communities resulting in reduced ‘colonization resistance’ [52], or be direct vectors [56, 57]. Further research into the microbiota of *Argulus* and other fish ectoparasites, and their pathogen vectoring capabilities, will be valuable for understanding their role in coinfection dynamics. Intriguingly, rainbow trout raised in constant light had a significantly lower microbiome diversity and, when challenged with *Argulus*, exhibited greater shifts in taxonomic composition, and possibly functional potential, compared to fish under regular light-dark regimes. Given the growing body of evidence for the importance of ‘healthy’ microbial communities [58] for effective host homeostasis and disease resistance [59, 60], characterising circadian disruption to microbiomes is important for understanding animal disease risks.

We demonstrate significant daily dynamicity in the skin microbiome of rainbow trout; a substantial proportion of bacteria genera exhibit rhythmic changes in relative abundance, suggesting a temporal structure to microbiome functional activity. Parasitic infection appears to perturb microbiome composition, and our data predict shifts in the timings of peak biosynthetic, degradative and energy generation pathway activity in the microbial community. Understanding of the functional importance to the host of commensal microbiota in teleost skin is still in its infancy [52], and 16S rRNA predictive functional analyses are only indicative of actual microbial activity [61]. However, we also find strongly contrasting taxonomic and functional profiles between the two timepoints used for full-scale metagenomic analysis, which further supports daily cycles in microbiome composition and functions. Temporal metatranscriptomic and/or metaproteomic profiling will be an important means to build upon our results and to confirm functional cycles and their potential significance to host health. Moreover, the impact of circadian disruption on other aspects of fish biology, such as hormone levels [62], diet/feeding patterns and behaviour [63] and their subsequent influence on microbiomes, requires further research. Nevertheless, as interest builds towards the utility of microbiome engineering strategies to promote health and productivity in aquaculture [23, 52, 64], we propose that a chronobiological understanding of fish microbiomes may be crucial for their effectiveness. The daily rhythms of both fish host immunity and their microbiome communities, for example, could be critical to uptake and establishment of probiotics treatments. Chronotherapeutics—the timed application of treatments and vaccines [65]—in human medicine holds great promise for improving efficacies but is yet to be given full consideration for managed animal health.

In the mammalian gut—by far the most studied host-microbiome interface—there is a complex interplay between immune factors that shape microbial communities, and, conversely, microbiota profoundly affecting immune system development and maintenance [14, 15]. Mammal gut microbiome daily rhythms may themselves play a role in host circadian health [66, 67]. However, in other tissues, and particularly for non-mammalian vertebrates, host immune-microbiome connectivity and circadian dynamics remain poorly understood. For teleosts, there is evidence that macrophages [68] and adaptive immune components (e.g. T cells [69] and immunoglobulins [70]) may be key to mucosal microbiome composition. Our study is the first to present an integrated analysis of skin microbiomes with a broad set of immune and circadian clock gene expression profiles in fish. We found genes of the core secondary feedback loops (e.g. *bmal*, *clock*, *rora*, *csnk1d*) that define the vertebrate molecular clock to be strongly associated with microbial taxa relative abundances in uninfected rainbow trout under 12:12 LD, yet these direct clock-microbe associations were largely absent in constant light. Similarly, mice faecal microbiota composition appears closely linked to *bmal1*, with knock-outs resulting in arrhythmicity and altered abundance of microbial taxa [17]. Our results suggest this arm of the biological clock may be pivotal to orchestrating changes in mucosal microbiomes across vertebrates. However, we also find perturbation of microbial communities via ectoparasite infection reconfigures the connectivity of host expression and microbiota. In both photoperiods, lice-infected fish immune-microbe networks show a greater level of connectivity between host immune gene expression and microbial taxa compared to uninfected individuals. In particular, our results indicate T cell markers to be central to this host-microbiome interface during ectoparasite infection. Under 12:12 LD, we find the T helper cell gene *cd4* to be strongly linked to microbiome composition, whilst in constant light the cytotoxic T cell marker *cd8a* appears to be more influential to microbiome-immune associations. For teleost fish, the ratios and distributions of T cell populations are not well defined [71, 72], although CD4+ and CD8+ subsets appear to have different roles in pathogen defence [73]. Our results suggest their relative importance to shaping fish mucosal microbiomes, or vice versa, warrant further investigation. Disentangling the directionality of the associations, we find via controlled manipulations of host immune cell populations [74, 75], clock gene expression [76] and microbiota [77] will undoubtedly be key to advancing the concept of circadian holobiont health.

Our study demonstrates the complex daily interaction of fish immune expression and microbiomes, which are impacted by photoperiod and infection status. There is rapidly growing recognition for the detrimental impacts of circadian rhythm perturbation in human medicine [13], though little attention has been paid to the implications for animal health. In an industry that heavily utilises light manipulation, contemporary aquaculture practices may be significantly exacerbating current disease issues. We provide here an important resource for furthering efforts to integrate chronobiology into animal disease mitigation strategies. In addition, as artificial light at night (i.e. light pollution) encroaches on ever greater proportions of the world’s ecosystems [78, 79], we propose that circadian disruption may have as yet undiscovered implications for health and disease dynamics in wild animal populations.

## Methods

### Experimental design and sample collection

Juvenile female triploid rainbow trout fry (*O. mykiss*, 10-day post-yolk sac absorption, *n* = 500) were obtained from a commercial hatchery (Bibury Trout Farm, UK). Fry were visually and microscopically determined free of parasitic infections upon arrival and maintained in a re-circulating aquaculture system (RAS) in Cardiff University (water temperature 12 ± 0.5 °C, pH 7.5 ± 0.2). The rainbow trout were randomly assigned to duplicate tanks (45 × 60 × 60 cm, 150 L) under one of two photoperiod conditions; 12:12 LD (lights on at zeitgeber time 0; ZT0, off at ZT12) or 24:0 LD (constant light, 24:0 LD). Each tank was individually illuminated with a full-spectrum white LED bar (80 lux at surface) and surrounded with blackout material to ensure no disturbance from ambient light. Fish were fed with a commercial trout feed (Nutraparr, Skretting, UK) ad libitum at ZT2-3 and ZT9-10 daily. Water oxygen saturation (> 90%), ammonia (< 0.02 mg/L), nitrite (< 0.01 mg L−1) and nitrate (< 15 mg L−1) were maintained within an appropriate range.

After 1 month acclimation to light conditions, 130 fish from each light treatment were individually isolated in 1 L plastic containers. Half of the fish from each light treatment (*n* = 65 per treatment) were individually inoculated with ten *Argulus foliaceus* metanauplii (24-h post-hatching). *Argulus* metanauplii were obtained from eggs of wild-caught adult pairs (sourced from Risca Canal, Newport), maintained at Cardiff University. Egg strings were collected and hatched under laboratory conditions according to Stewart et al. (2018). Inoculations were performed at ZT4-5. Fish were individually held in a glass container with 50 ml of tank water and 10 metanauplii added. Fish were observed until all lice had attached (within 2 min) and then returned to their 1 L container. Control fish (those not inoculated with *Argulus* lice) were also held for 2 min in 50 ml of water to control for handling stress. Water in all individual containers were changed daily, feeding continued on schedule outlined above, and light conditions maintained at same intensity, spectrum and duration as during acclimation period. The remaining fish were maintained in the RAS system. Once a week, 30 random fish per light treatment were weighed (g) and measured (standard length, SL in cm) for 16 weeks to monitor growth rates. General linear models of standard length and weight, including photoperiod and sampling day, were used to assess differences in growth between light treatments. All procedures were performed under Home Office project licence PPL 303424 with full approval of Cardiff University Animal Ethics committee.

One week after inoculation, sampling of fish was performed over a 48-h period to encompass two full circadian cycles. Starting at ZT0 (lights on in 12:12 LD treatment), every 4 h, five fish from each condition (12:12 LD control, 12:12 LD *Argulus*-infected, 24:0 LD control, 24:0 LD *Argulus*-infected) were euthanised using an overdose of tricaine methanesulfonate (MS222, 500 mg L^− 1^) according to Home Office Schedule 1. At timepoints during dark periods in 12:12 LD treatment, fish were handled and euthanised in dim red light. Immediately after euthanasia, infected fish were visually inspected to quantify number of lice surviving and the lice removed to ensure they were not included in tissue samples. Welch’s two sample *T* test was used to determine difference in infection load (number of *Argulus*) between light treatments. All sampled fish were weighed (g) and measured (standard length, SL in cm). Skin swabs (MWE MW-100) were rubbed along the entire lateral body surface five times each side and immediately frozen at −80 C to preserve skin mucus microbiota for DNA extraction. All skin from immediately posterior to opercula to the caudal peduncle was dissected using sterile forceps, preserved in RNAlater (Invitrogen), and stored at −80 °C until RNA extraction. All dissections for each timepoint were performed within an hour window. At each timepoint-treatment combination, 10 ml of water from all containers was pooled and frozen at −80 °C to provide background controls for skin microbiome analyses. To test for endogenous expression rhythms, an additional 65 uninfected fish maintained at 12:12 LD were individually isolated and held in constant darkness (DD). After 24 h, starting at ZT0, five fish every 4 h were sampled as above.

### RNA extraction, gene expression quantification and analyses

Total RNA was individually extracted from each skin sample using RNeasy Mini kits (Qiagen). RNA was quantified using Qubit Broad Range RNA assays (ThermoFisher Scientific). mRNA expression patterns in the skin were measured by Nanostring analysis, following manufacturer’s guidelines, at Liverpool Centre for Genomic Research. The nCounter PlexSet oligonucleotide and probe design was performed at NanoString Technologies (NanoString Technologies) for 48 genes, including four housekeeping genes (Supplementary Table 9). The oligonucleotide probes were synthesised at Integrated DNA Technologies. Titration reactions were performed according to supplier’s instructions with RNA inputs between 250 and 700 ng to determine the required RNA amount for hybridization reaction. Six hundred nanograms of total RNA per sample was used for PlexSet hybridization reaction for 20 h according to manufacturer’s instructions.

Samples were processed on a nCounter MAX prep station (NanoString Technologies) and cartridges were scanned in a generation II nCounter Digital Analyzer (NanoString Technologies). RCC files (nCounter data files) were used for data analysis. RCC files were imported into the NanoString nSolver 4.0 analysis software and raw data pre-processing and normalisation was performed according to manufacturer’s instructions for standard procedures (positive normalisation to geomean of top 3 positive controls, codeset content normalisation using housekeeping genes *hprt1*, *polr1b*, *polr2i*, and codeset calibration with the reference sample). The housekeeping gene *rplp0* and *aanat2* expression were not detected and excluded from analyses.

To assess overall differences in immune responses to infection under the different light treatments, pairwise *t* tests comparing normalised expression of immune genes were performed in R (version 4.0.3). To detect rhythmicity in expression of clock and immune genes, empirical JTK Cycle (eJTK_cycle [80]) analyses were applied with a set period of 24 h, a phase search every 4 h from ZT0 to ZT20, and an asymmetry search every 4 h from ZT4 to ZT20. FDR-corrected empirical *p* values less than 0.1 were considered moderately rhythmic [81–83], and less than 0.05 strongly rhythmic. CircaCompare [31] was used to estimate rhythmic genes’ peak expression time, mesor and amplitude, and to statistically contrast rhythms.

### DNA extraction, 16S rRNA gene metabarcoding and metagenomic analyses

DNA was extracted from skin swabs using Qiagen DNeasy Blood and Tissue kits according to Gill et al. [84] to maximise lysis of microbiome community and DNA recovery. PCR amplification of the 16S rRNA V4 region, using 515F and 806R primers, was performed in triplicate for each DNA extract, pooled and prepared for Illumina MiSeq sequencing according to Caporaso et al. [85] (1-step PCR 16S amplification and incorporation of Illumina adapters/indexes). Gel electrophoresis was used to estimate concentrations for pooling individual amplicon libraries. Negative controls for extractions and PCR, and mock community positive (HM-783D, BEI Resources) controls were included for sequencing. Libraries were sequenced using a 2 × 250 bp Illumina MiSeq v2 run at the Cardiff University School of Biosciences Genomics Hub.

Paired-end demultiplexed Illumina sequencing reads were imported into the Quantitative Insights Into Microbial Ecology 2 (QIIME2) [86]. Sequences were then quality filtered, dereplicated, chimaeras identified and paired-end reads merged in QIIME2 using DADA2 [87] with default settings (--p-trunc-len-f 225, --p-trunc-len-r 196, --p-max-ee-f/r 2, --p-trunc-q 2, minimum overlap = 12 bp, no mismatch). Classification of amplicon sequence variants (ASVs) was performed using a Naïve Bayes algorithm trained using sequences representing the bacterial V4 rRNA region available from the SILVA database (https://www.arb-silva.de/download/archive/qiime; Silva_132), and the corresponding taxonomic classifications were obtained using the q2-feature-classifier plugin in QIIME2. The classifier was then used to assign taxonomic information to representative sequences of each ASV. Following rarefaction analysis, samples with less than 2000 sequences were excluded from further analyses. QIIME2 was used to analyse alpha (Kruskal-Wallis pairwise tests of Faith’s phylogenetic distance) and beta (pairwise PERMANOVA) diversity measures. ASVs were filtered to exclude those assigned to eukaryotes or eukaryotic organelles and include ones with at least 100 copies in at least two samples. The QIIME2 output data were imported in RStudio (version 1.3.959) with the Bioconductor package phyloseq [88], for subsetting, normalising and plotting of the data. No reads passed data pre-processing and filtering in extraction blanks and PCR negative controls. The mock community positive control profile matched the manufacturer’s expected composition and relative abundances (data not shown).

Differential abundance of ASVs between healthy and infected fish in both light treatments was determined using DESeq2 [89], with FDR-corrected *p* values less than 0.05 considered significant. Differential abundances of all taxonomic levels were also determined and visualised using MicrobiomeAnalyst [90] heat trees [91] using default settings. We inferred the microbial gene content from the taxa abundance using PICRUSt2 [39]. We used LefSe analyses to identify group differences in the inferred gene abundance of MetaCyc pathways, using the online galaxy server (https://huttenhower.sph.harvard.edu/galaxy/). LDA scores > 2.0 were considered significant. Rhythmicity of microbial genera and MetaCyc pathway abundances were determined following the same methods as gene expression (see above). To determine potential associations of host gene expression and the microbiome, Spearman correlation tests (R package Hmisc rcorr function) were performed including only genera found in at least 50% of samples in each treatment group. Corrected *p* values (using qvalue R package FDR correction) of less than 0.05 were considered significantly correlated. Correlation networks were visualised using gephi [92] (with Force Atlas2 algorithm) and influential nodes determined using degree centrality scores and number of connections (degrees).

A subset of 12 samples (3 healthy and 3 infected individuals under 12:12 LD from two timepoints; onset of light and onset of dark, randomly selected) were prepared for full metagenomic sequencing. To reduce host DNA, aliquots of swab extracts (the same used for 16S rRNA profiling) were prepared using the NEBNext Microbiome Enrichment Kit (New England Biolabs) according to manufacturer instructions. The microbial-enriched DNA (6-24 ng input per sample) was then prepared for sequencing using the Illumina DNA Prep Kit (Illumina) according to manufacturer instructions. Libraries were quantified and pooled equimolarly, based on Qubit HS DNA assays. Library quality was checked using Agilent Tapestation D1000 assays. Indexed libraries were sequenced on a high-throughput 2 × 150 bp NextSeq 550 run at Cardiff University School of Biosciences Genomics Hub.

Adapter removal and quality trimming of raw metagenomic reads was performed using Trimmomatic v0.39 [93] with default parameters except increased quality score thresholds of Q30. Duplicate reads were removed using seqkit [94] and paired reads repaired using BBtools [95]. Deduplicated reads were filtered against the rainbow trout genome [96] using minimap2 [97]. Paired and single filtered reads from all samples (78.85 Gb, average 6.57 Gb per sample) were used for metagenome co-assembly in Megahit v1.2.9 [98] using meta-sensitive pre-set. Sample reads were individually mapped to assembled contigs using bowtie2 [99] with default parameters, and contigs were binned using Metabat2 v2.15 [100]. Metagenomic assembled genomes (MAGs) were checked for quality using CheckM [101] and refined using Anvi’o 7 [102]. MAG phylotyping was performed using Amphora2 [103], with phylotype assigned to lowest taxonomic level with at least 75% agreement in markers. Species trees, using all single-copy orthologs, were generated for selected MAGs (bin 3 *Flavobacterium* sp., bin 11 *Pseudomonas* sp.), against other named species within their genus with complete genomes (*Flavobacterium* = 33, *Pseudomonas* = 92), using Orthofinder [104]. Differential MAG abundance between treatment groups was determined using DESeq2 [89] with FDR-corrected *p* value threshold < 0.05. Trimmed reads were also aligned against the NCBI nr protein database using Diamond [105] and imported into Megan 6 [106] for taxonomic profiling and functional assignments. Raw counts of SEED subsystems were imported into R for normalisation and differential abundance testing using DESeq2 (FDR-corrected *p* value threshold < 0.05).

## Supplementary Information

**Additional file 1: Supplementary Table 1**. Gene rhythm summary

**Additional file 2: Supplementary Table 2**. AlphaDiv

**Additional file 3: Supplementary Table 3**. BetaDiv

**Additional file 4: Supplementary Table 4**. MAG_DeSeq2

**Additional file 5: Supplementary Table 5**. 16S_DeSeq2

**Additional file 6: Supplementary Table 6**. ArgvCtl_Lefse

**Additional file 7: Supplementary Table 7**. SEEDlvl3_DeSeq2

**Additional file 8: Supplementary Table 8**. mic rhythm summary

**Additional file 9: Supplementary Table 9**. HostGenes

**Additional file 10: Supplementary Figure 1.** Average A) standard length and B) weight of trout (±1 S.E.) over 16-week growth trial under 12:12 LD (orange) and 24:0 LD (yellow). C) Boxplots of number of Argulus foliaceus lice infecting fish 7 days post-inoculation. **Supplementary Figure 2.** Mean expression (± 1 S.E.) of accessory clock genes of uninfected (cyan) and Argulus-infected (orange) rainbow trout maintained at 12:12 LD (left) and 24:0 LD (LL, right). Expression is normalised counts of mRNA copies detected via Nanostring nCounter. Curves denote cosinor waveform fitted using CircaCompare. Grey shading indicates time periods in darkness (grey dashing indicates equivalent 12:12 LD light transitions on LL plots). **Supplementary Figure 3.** Mean expression (± 1 S.E.) of clock genes of rainbow trout under 12:12 LD and DD (free-running, constant darkness). Expression is normalised counts of mRNA copies detected via Nanostring nCounter. Curves denote cosinor waveform fitted using CircaCompare. Grey shading indicates time periods in darkness (grey dashing indicates subjective day-night transition in DD). **Supplementary Figure 4.** Mean expression (± 1 S.E.) of innate immune genes of uninfected (cyan) and Argulus-infected (orange) rainbow trout maintained at 12:12 LD (left) and 24:0 LD (LL, right). Expression is normalised counts of mRNA copies detected via Nanostring nCounter. Curves denote cosinor waveform fitted using CircaCompare. Grey shading indicates time periods in darkness (grey dashing indicates equivalent 12:12 LD light transitions on LL plots). Only genes with significant rhythm in one or more groups shown. **Supplementary Figure 5.** Mean expression (± 1 S.E.) of adaptive immune genes of uninfected (cyan) and Argulus-infected (orange) rainbow trout maintained at 12:12 LD (left) and 24:0 LD (LL, right). Expression is normalised counts of mRNA copies detected via Nanostring nCounter. Curves denote cosinor waveform fitted using CircaCompare. Grey shading indicates time periods in darkness (grey dashing indicates equivalent 12:12 LD light transitions on LL plots). Only genes with significant rhythm in one or more groups shown. **Supplementary Figure 6.** Mean expression (± 1 S.E.) of immune genes of rainbow trout under 12:12 LD and DD (free-running, constant darkness). Expression is normalised counts of mRNA copies detected via Nanostring nCounter. Curves denote cosinor waveform fitted using CircaCompare. Grey shading indicates time periods in darkness (grey dashing indicates subjective day-night transition in DD). **Supplementary Figure 7.** A) Rarefaction plots of detected amplified sequence variants (ASVs) by sampling depth. B) NMDS ordination of microbiome profiles. C) Alpha diversity plots by treatment group. Argulus_12 = Argulus-infected rainbow trout under 12:12 LD, Argulus_24 = Argulus-infected rainbow trout under 24:0 LD, Control_12 = healthy rainbow trout under 12:12 LD, Control_24 = healthy rainbow trout under 24:0 LD. **Supplementary Figure 8.** Comparison of rainbow trout skin microbiome taxonomic profiles between 16S rRNA metabarcoding (taxonomic assignment of ASVs in Qiime2) and metagenomic sequencing (taxonomic assignment of reads in Megan). **Supplementary Figure 9.** Left: Heatmap of MAG relative abundance (scaled to median), yellow indicates increased abundance, blue indicates reduced abundance. Supplementary table 4 provides significant differences among treatment group. Right: Examples of MAGs found to be differentially abundant between infection status and/or timepoint. **Supplementary Figure 10.** Species tree of Pseudomonas genomes generated by OrthoFinder. MAG from current study highlighted in bold. **Supplementary Figure 11.** Species tree of Flavobacterium genomes generated by OrthoFinder. MAG from current study highlighted in bold. **Supplementary Figure 12.** Co-occurrence networks of microbial genera (pink) and host gene expression (orange = clock, green = immune, blue = corticotropin) in healthy (top) and Argulus-infected (bottom) trout under 24:0 LD. Node and label size scaled to degree centrality score. Label colour denotes rhythmicity (black = rhythm FDR *p*-value <0.05, grey = rhythm FDR p-value >0.05). Connection colour indicates association (grey = positive, red = negative, determined by Spearman correlation tests) and connection width scaled to correlation strength (thicker lines denote a higher correlation coefficient).

**Additional file 11.** Supplementary Datafile 1

## Data Availability

16S rRNA gene sequence data are submitted to the NCBI Sequence Read Archive (SRA) under accession PRJNA694669. Metagenomic sequence data are submitted to NCBI SRA under accession PRJNA757611. All other datasets analysed during the current study and their associated scripts are available from the corresponding author on request.
