# Supplementary material for: Circadian dynamics of the teleost skin immune-microbiome interface
Source: Microbiome. 2021 Nov 16;9:222. doi: 10.1186/s40168-021-01160-4 (PMC8594171; doi:10.1186/s40168-021-01160-4)
Supplement: Supplementary file 3 — Additional file 2: Supplementary Table 2. AlphaDiv [file 40168_2021_1160_MOESM3_ESM.pdf]

Group 1	Group 2	H	p-value
Argulus_12	Argulus_24	0.052886766	0.818114126
Argulus_12	Control_12	0.509925111	0.475171277
Argulus_12	Control_24	13.50547798	0.000237868
Argulus_12	Water	32.64	1.10909E-08
Argulus_24	Control_12	0.10845712	0.741907566
Argulus_24	Control_24	14.01007431	0.000181834
Argulus_24	Water	31.85643414	1.65999E-08
Control_12	Control_24	12.92404919	0.000324387
Control_12	Water	34.22222222	4.91643E-09
Control_24	Water	33.81818182	6.05107E-09

q-value
0.818114126
0.593964096
0.000396447
3.69695E-08
0.818114126
0.000363668
4.14997E-08
0.000463411
3.02553E-08
3.02553E-08
