# Supplementary material for: Circadian dynamics of the teleost skin immune-microbiome interface
Source: Microbiome. 2021 Nov 16;9:222. doi: 10.1186/s40168-021-01160-4 (PMC8594171; doi:10.1186/s40168-021-01160-4)
Supplement: Supplementary file 4 — Additional file 3: Supplementary Table 3. BetaDiv [file 40168_2021_1160_MOESM4_ESM.pdf]

Group 1	Group 2	pseudo-F	p-value	q-value
Argulus_12	Argulus_24	1.927441837	0.004	0.004
Argulus_12	Control_12	3.167869012	0.001	0.001111111
Argulus_12	Control_24	4.887142676	0.001	0.001111111
Argulus_12	Water	14.804493	0.001	0.001111111
Argulus_24	Control_12	6.276399245	0.001	0.001111111
Argulus_24	Control_24	7.37383445	0.001	0.001111111
Argulus_24	Water	15.17727828	0.001	0.001111111
Control_12	Control_24	3.307888287	0.001	0.001111111
Control_12	Water	17.5535308	0.001	0.001111111
Control_24	Water	19.88133954	0.001	0.001111111
