# Supplementary material for: Circadian dynamics of the teleost skin immune-microbiome interface
Source: Microbiome. 2021 Nov 16;9:222. doi: 10.1186/s40168-021-01160-4 (PMC8594171; doi:10.1186/s40168-021-01160-4)
Supplement: Supplementary file 10 — Additional file 9: Supplementary Table 9. HostGenes [file 40168_2021_1160_MOESM10_ESM.pdf]

Gene	Genbank Accession	RefSeq Gene ID	
clock1a	GU228520	100135915	Clock genes
clock1b	GU228521	110521676	
clock3	GU228522	110533857	
bmal1	GQ489026	100499618	
bmal2	CX717649	110499965	
per1	AF228695	100135905	
per2		110508801	
cry1	CA383214	110522881	
cry2		110499866	
reverbb	AF342943	100135954	
aanat2	AF106006	100135881	
rora		110520931	
csnk1d		110499233	
timeless		110494196	
il1b	AJ223954	100136024	Immune genes
il4	FN820501	100653462	
il6	DQ866150	100136689	
il10	AB118099	100136835	
il17a	AJ580842	100136642	
tnfa	AJ277604	100136034	
nos2	AJ295230	100136036	
ifng	AJ616215	100136643	
tgfb	AJ007836	100136774	
tcrb	AJ517930	110504270	
igt	AY870263		
igm	X65261		
igd	AY870262		
tbx21	FM863825	100500940	
gata3	FM863826	100500939	
foxp3b	FM883711	100653438	
rory	FM883712	100528059	
cd4	AY973030	100136285	
cd8a	AF178053	100135889	
cath1	AY594646	100136204	
cath2	AY542963	100136187	
hamp	AF281354	100135935	
tlr2	HE979560	100750259	
tlr9	EU627195	100170212	
tlr22	AJ628348	100136113	
mhcii	AY273808	100500791	
chi	AJ535688	100136076	
c3	L24433	110489027	
crf	AY049980	100135941	Corti
pomc	X69808	100136771	
rplp0		110494133	Controls
polr2i		100305160	
hprt1		110504699	
polr1b		110526594	
