# Supplementary material for: Circadian dynamics of the teleost skin immune-microbiome interface
Source: Microbiome. 2021 Nov 16;9:222. doi: 10.1186/s40168-021-01160-4 (PMC8594171; doi:10.1186/s40168-021-01160-4)
Supplement: Supplementary file 11 — Additional file 10: Supplementary Figure 1. Average A) standard length and B) weight of trout (±1 S.E.) over 16-week growth trial under 12:12 LD (orange) and 24:0 LD (yellow). C) Boxplots of number of Argulus foliaceus lice infecting fish 7 days post-inoculation. Supplementary Figure 2. Mean expression (± 1 S.E.) of accessory clock genes of uninfected (cyan) and Argulus-infected (orange) rainbow trout maintained at 12:12 LD (left) and 24:0 LD (LL, right). Expression is normalised counts of mRNA copies detected via Nanostring nCounter. Curves denote cosinor waveform fitted using CircaCompare. Grey shading indicates time periods in darkness (grey dashing indicates equivalent 12:12 LD light transitions on LL plots). Supplementary Figure 3. Mean expression (± 1 S.E.) of clock genes of rainbow trout under 12:12 LD and DD (free-running, constant darkness). Expression is normalised counts of mRNA copies detected via Nanostring nCounter. Curves denote cosinor waveform fitted using CircaCompare. Grey shading indicates time periods in darkness (grey dashing indicates subjective day-night transition in DD). Supplementary Figure 4. Mean expression (± 1 S.E.) of innate immune genes of uninfected (cyan) and Argulus-infected (orange) rainbow trout maintained at 12:12 LD (left) and 24:0 LD (LL, right). Expression is normalised counts of mRNA copies detected via Nanostring nCounter. Curves denote cosinor waveform fitted using CircaCompare. Grey shading indicates time periods in darkness (grey dashing indicates equivalent 12:12 LD light transitions on LL plots). Only genes with significant rhythm in one or more groups shown. Supplementary Figure 5. Mean expression (± 1 S.E.) of adaptive immune genes of uninfected (cyan) and Argulus-infected (orange) rainbow trout maintained at 12:12 LD (left) and 24:0 LD (LL, right). Expression is normalised counts of mRNA copies detected via Nanostring nCounter. Curves denote cosinor waveform fitted using CircaCompare. Grey shading indicates time pe [file 40168_2021_1160_MOESM11_ESM.pdf]

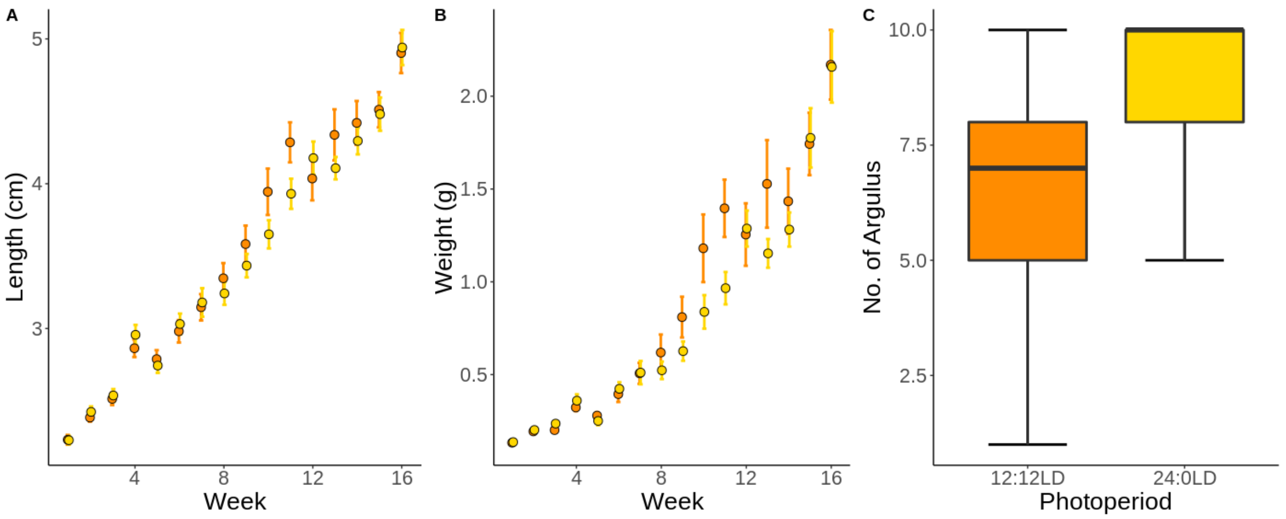

Supplementary Figure 1: Average A) standard length and B) weight of trout (±1 S.E.) over 16-week growth trial under 12:12 LD (orange) and 24:0 LD (yellow). C) Boxplots of number of *Argulus foliaceus* lice infecting fish 7 days post-inoculation.

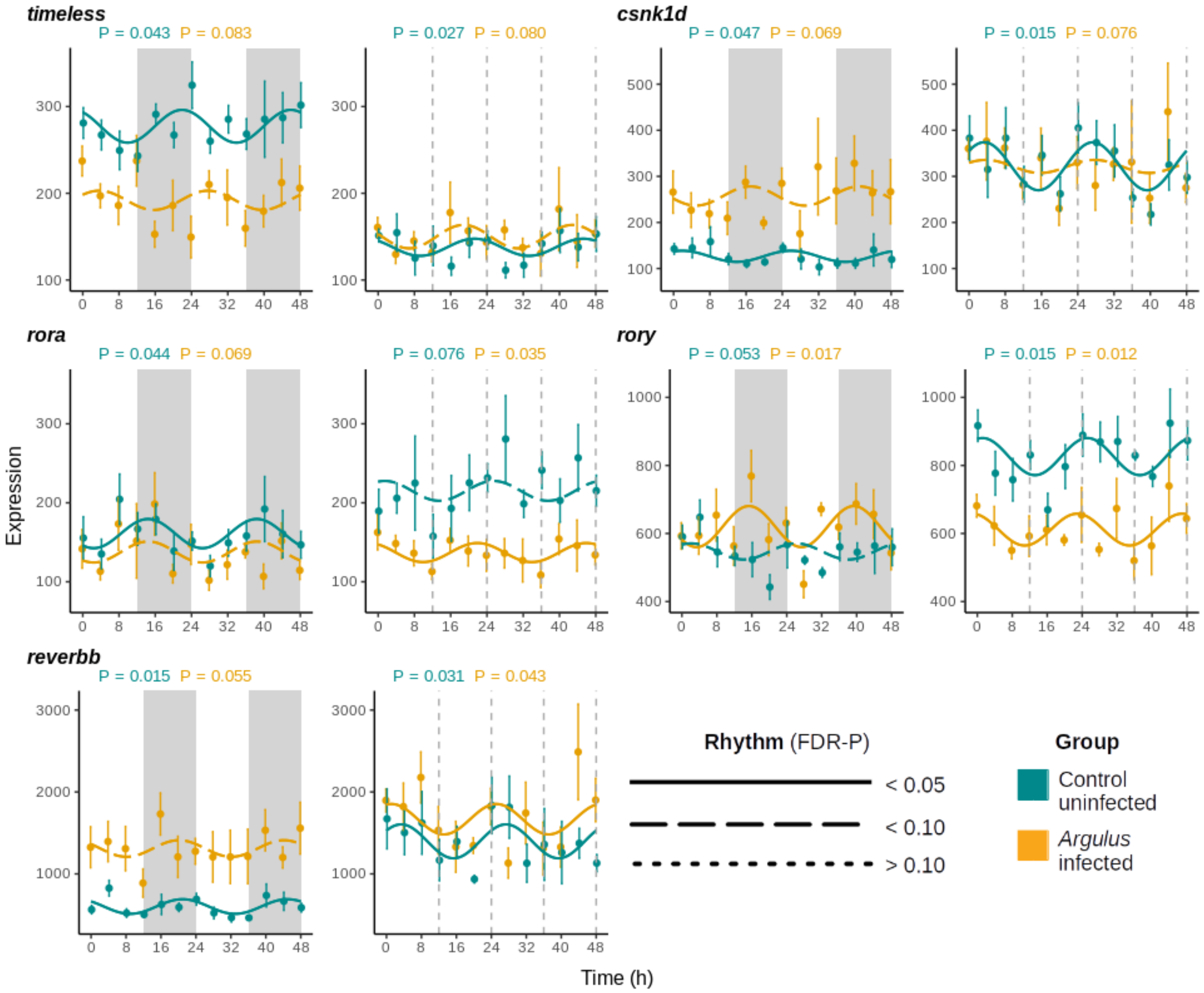

Supplementary Figure 2: Mean expression (± 1 S.E.) of accessory clock genes of uninfected (cyan) and *Argulus*-infected (orange) rainbow trout maintained at 12:12 LD (left) and 24:0 LD (LL, right). Expression is normalised counts of mRNA copies detected via Nanostring nCounter. Curves denote cosinor waveform fitted using CircaCompare. Grey shading indicates time periods in darkness (grey dashing indicates equivalent 12:12 LD light transitions on LL plots).

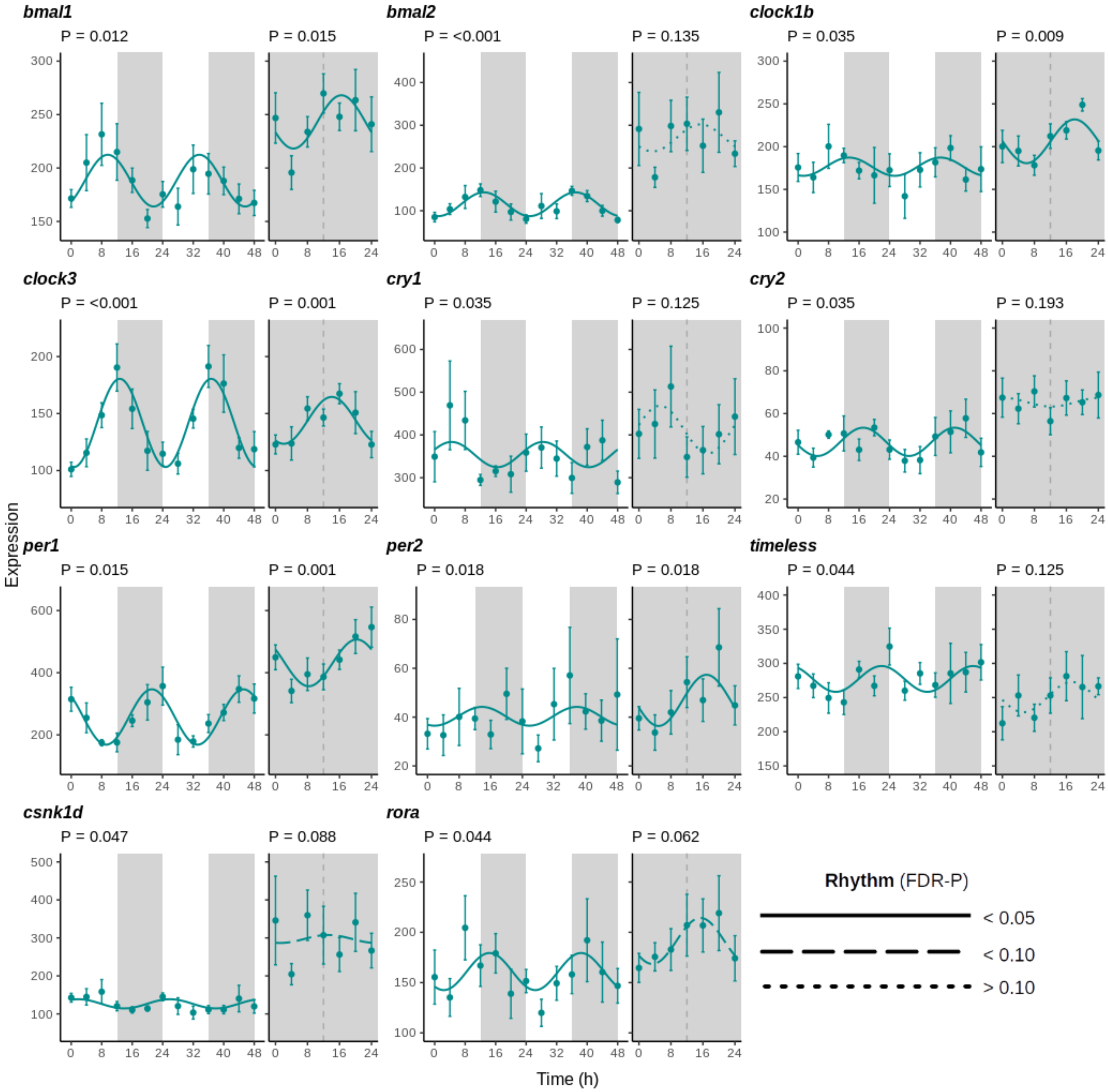

Supplementary Figure 3: Mean expression (± 1 S.E.) of clock genes of rainbow trout under 12:12 LD and DD (free-running, constant darkness). Expression is normalised counts of mRNA copies detected via Nanostring nCounter. Curves denote cosinor waveform fitted using CircaCompare. Grey shading indicates time periods in darkness (grey dashing indicates subjective day-night transition in DD).

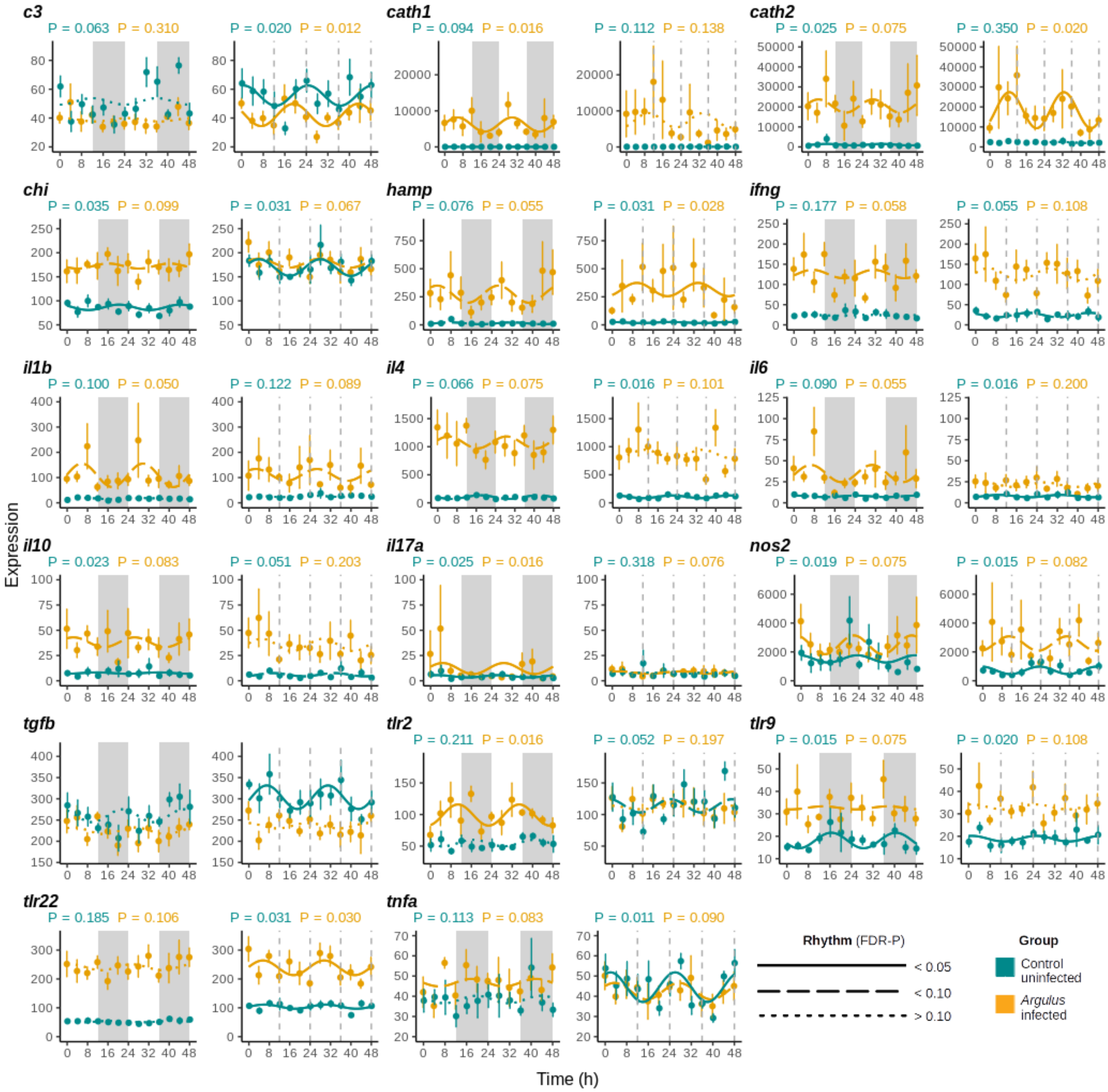

Supplementary Figure 4: Mean expression (± 1 S.E.) of innate immune genes of uninfected (cyan) and *Argulus*-infected (orange) rainbow trout maintained at 12:12 LD (left) and 24:0 LD (LL, right). Expression is normalised counts of mRNA copies detected via Nanostring nCounter. Curves denote cosinor waveform fitted using CircaCompare. Grey shading indicates time periods in darkness (grey dashing indicates equivalent 12:12 LD light transitions on LL plots). Only genes with significant rhythm in one or more groups shown.

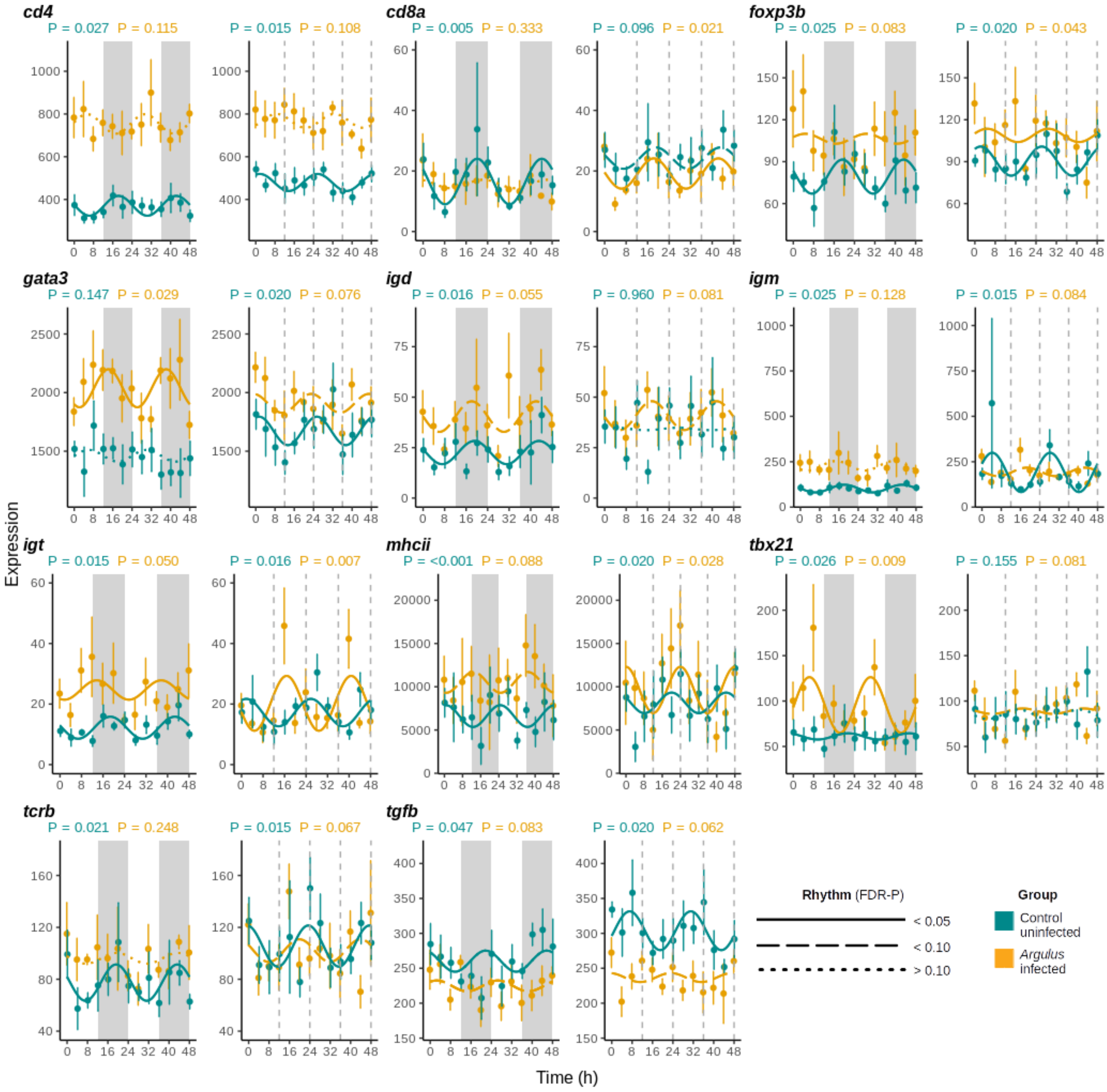

Supplementary Figure 5: Mean expression (± 1 S.E.) of adaptive immune genes of uninfected (cyan) and Argulus-infected (orange) rainbow trout maintained at 12:12 LD (left) and 24:0 LD (LL, right). Expression is normalised counts of mRNA copies detected via Nanostring nCounter. Curves denote cosinor waveform fitted using CircaCompare. Grey shading indicates time periods in darkness (grey dashing indicates equivalent 12:12 LD light transitions on LL plots). Only genes with significant rhythm in one or more groups shown.

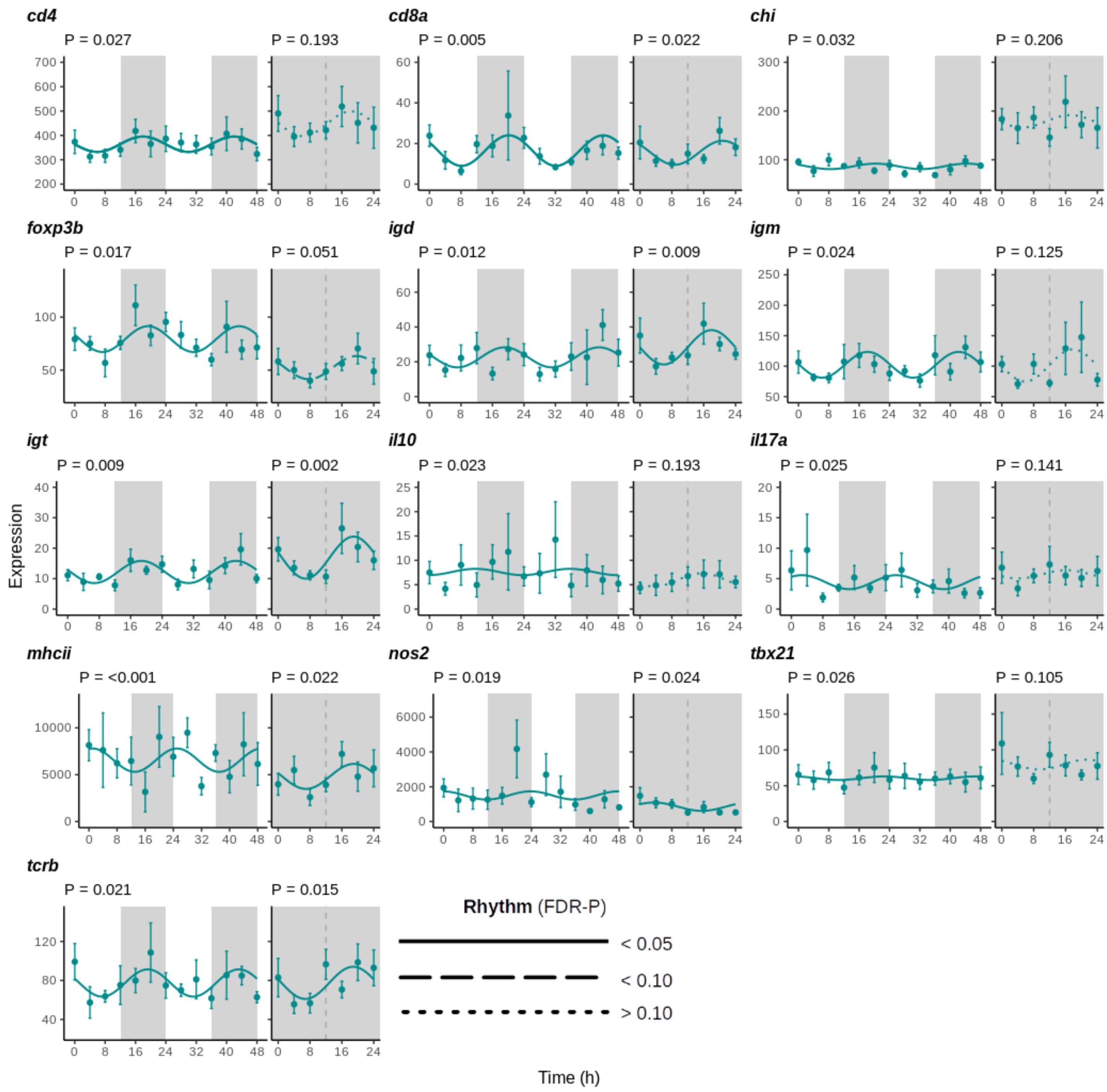

Supplementary Figure 6: Mean expression (± 1 S.E.) of immune genes of rainbow trout under 12:12 LD and DD (free-running, constant darkness). Expression is normalised counts of mRNA copies detected via Nanostring nCounter. Curves denote cosinor waveform fitted using CircaCompare. Grey shading indicates time periods in darkness (grey dashing indicates subjective day-night transition in DD).

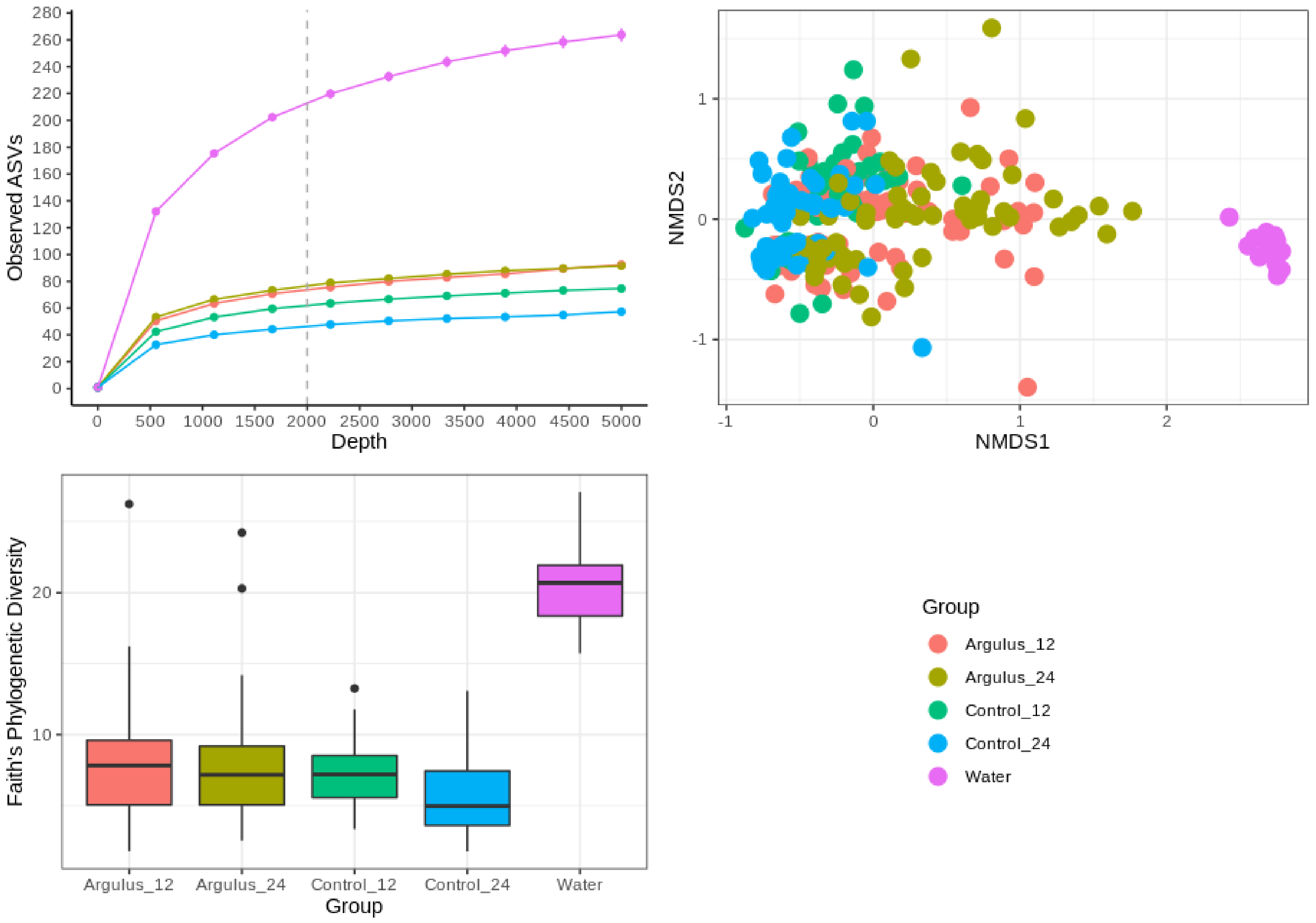

Supplementary Figure 7: A) Rarefaction plots of detected amplified sequence variants (ASVs) by sampling depth. B) NMDS ordination of microbiome profiles. C) Alpha diversity plots by treatment group. Argulus_12 = Argulus-infected rainbow trout under 12:12 LD, Argulus_24 = Argulus-infected rainbow trout under 24:0 LD, Control_12 = healthy rainbow trout under 12:12 LD, Control_24 = healthy rainbow trout under 24:0 LD.

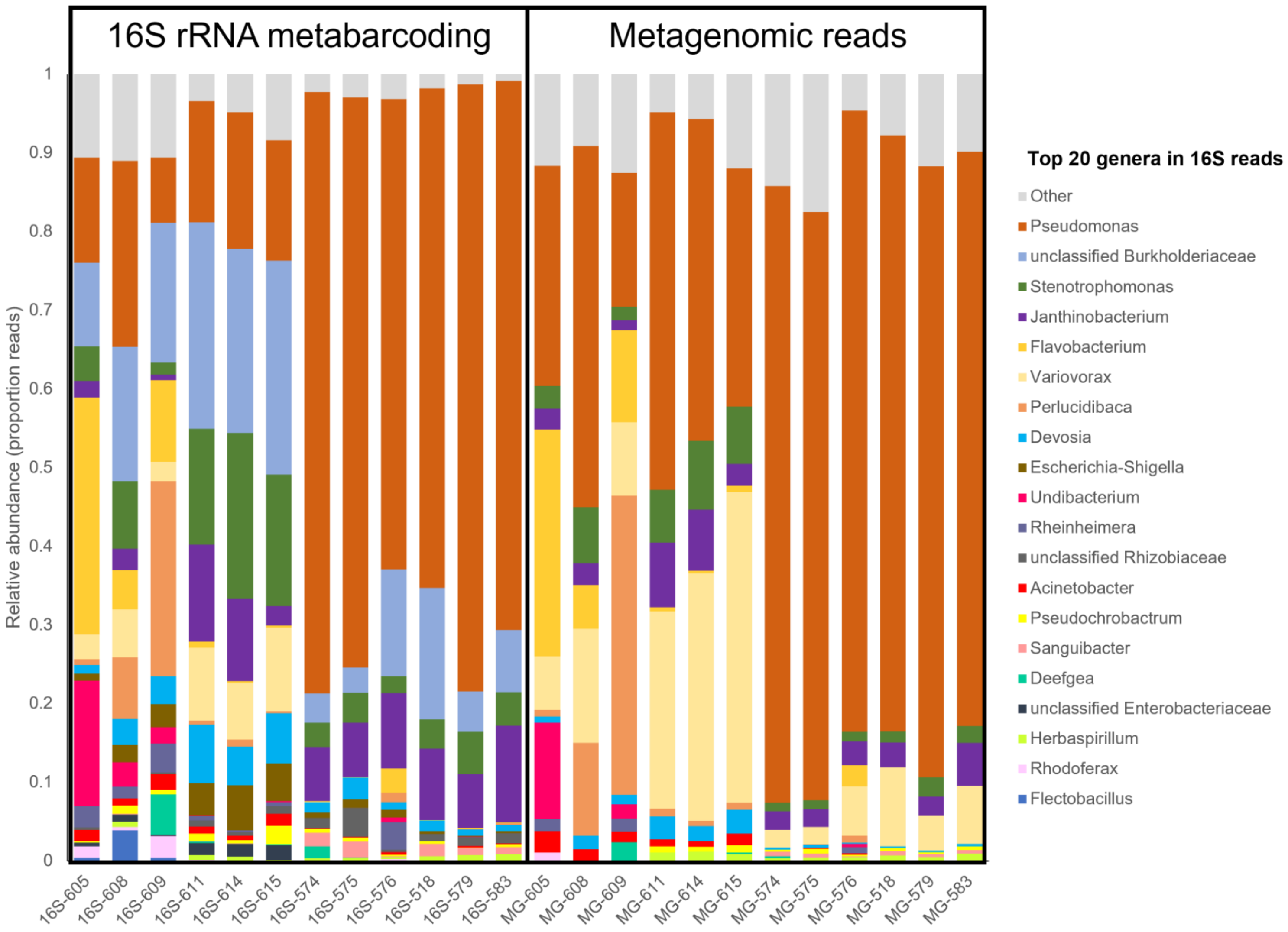

Supplementary Figure 8: Comparison of rainbow trout skin microbiome taxonomic profiles between 16S rRNA metabarcoding (taxonomic assignment of ASVs in Qiime2) and metagenomic sequencing (taxonomic assignment of reads in Megan).

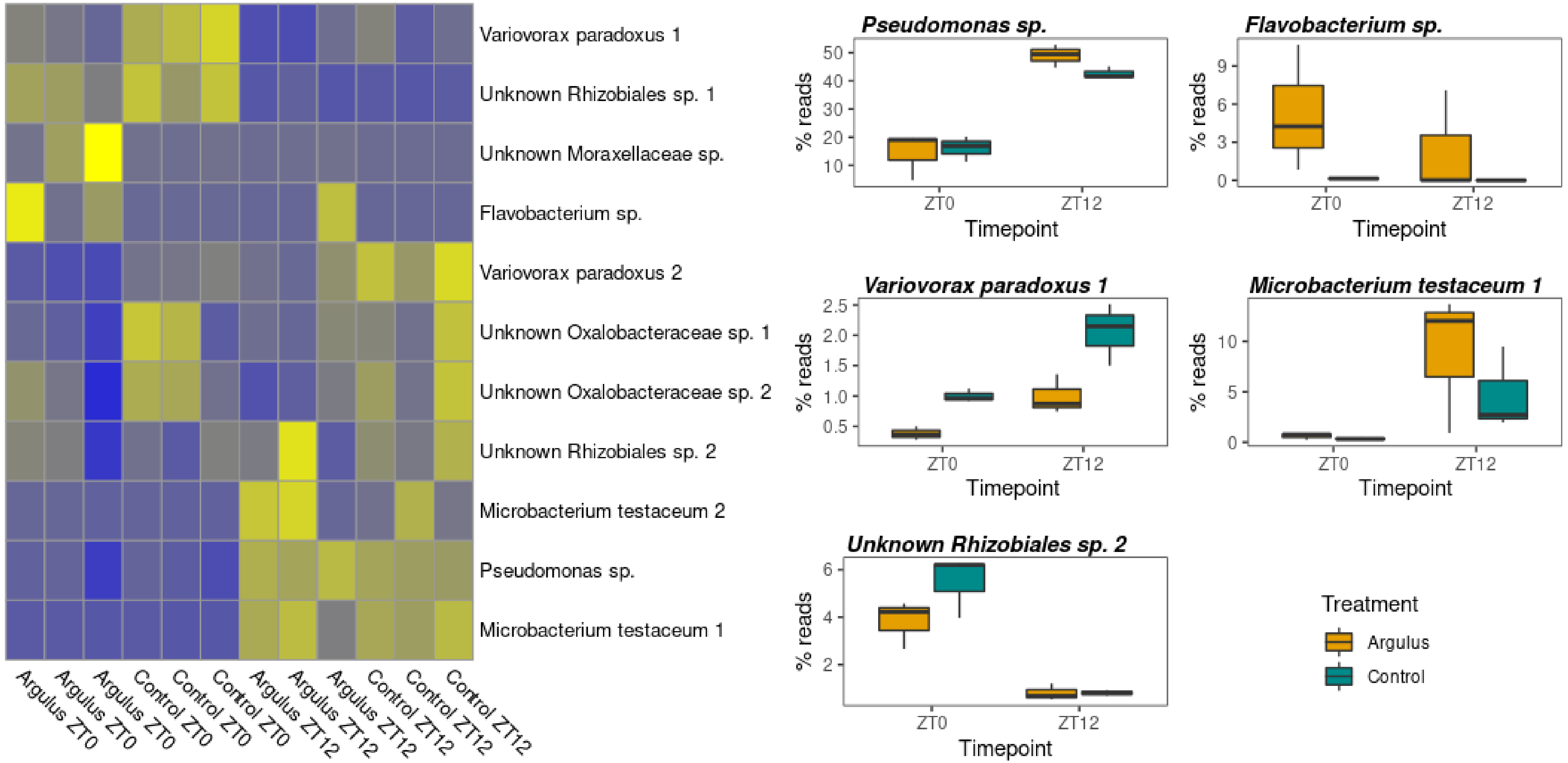

Supplementary Figure 9: Left: Heatmap of MAG relative abundance (scaled to median), yellow indicates increased abundance, blue indicates reduced abundance. Supplementary table 4 provides significant differences among treatment group. Right: Examples of MAGs found to be differentially abundant between infection status and/or timepoint.

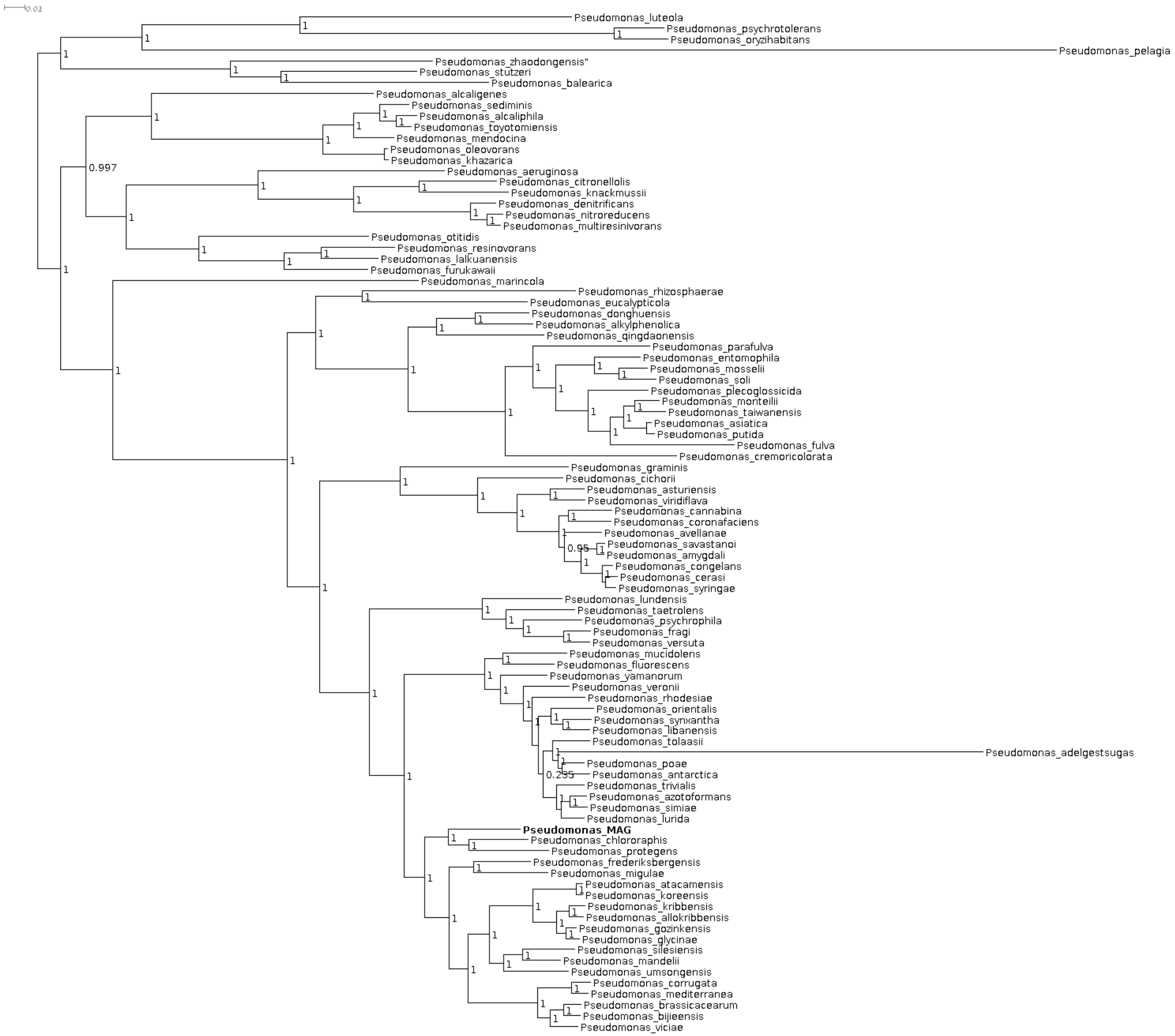

Supplementary Figure 10: Species tree of *Pseudomonas* genomes generated by OrthoFinder. MAG from current study highlighted in bold.

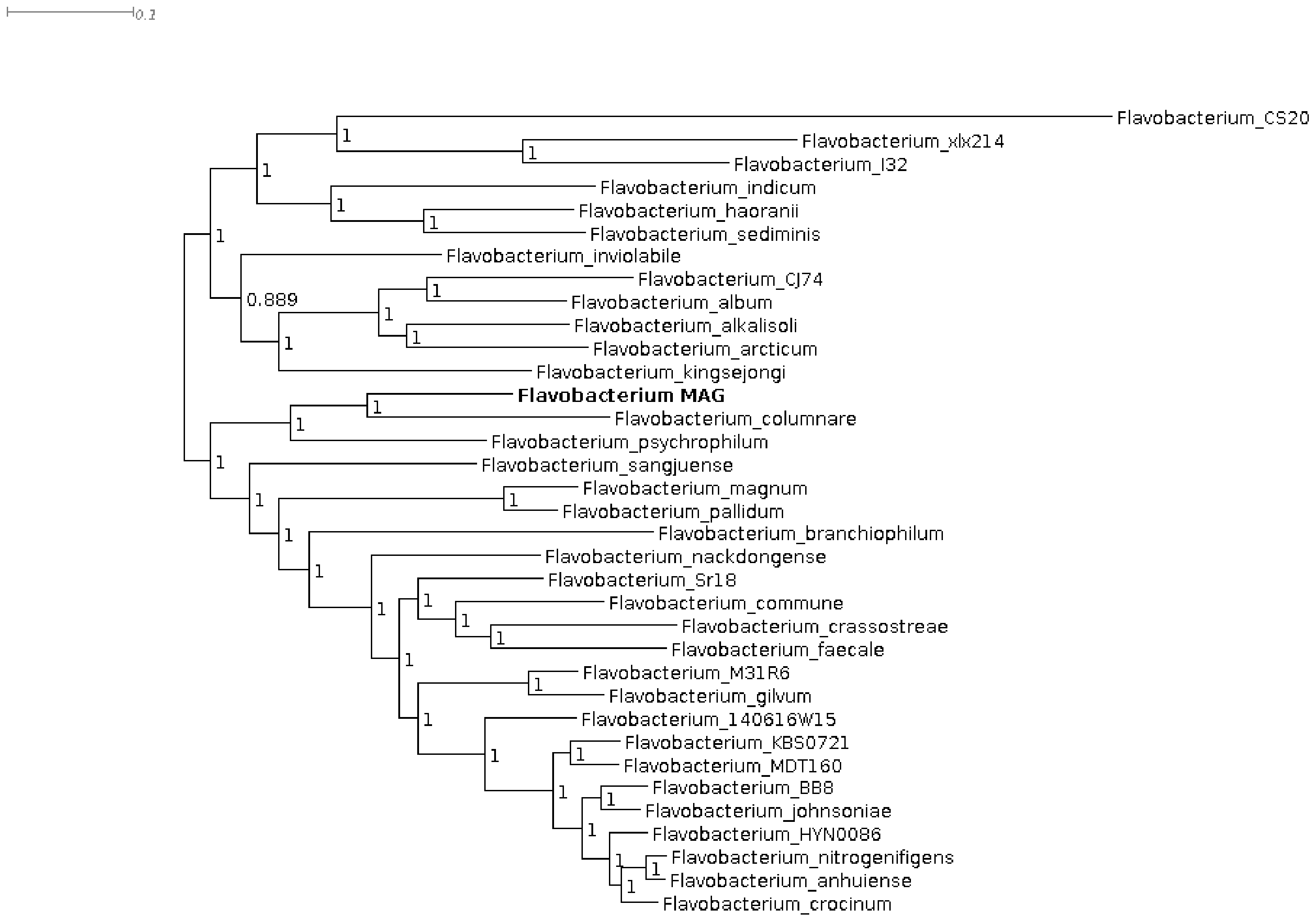

Supplementary Figure 11: Species tree of *Flavobacterium* genomes generated by OrthoFinder. MAG from current study highlighted in bold.

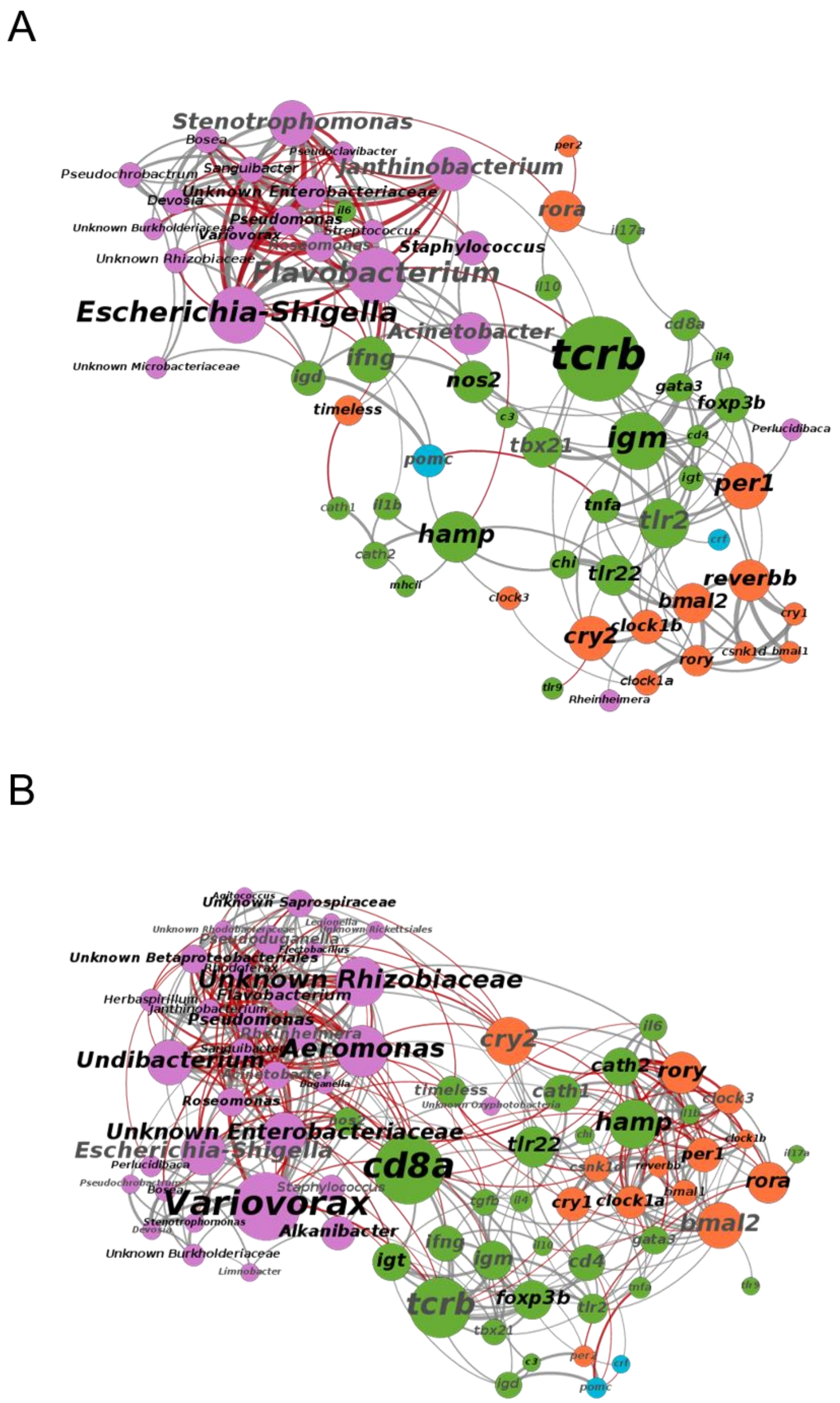

Supplementary Figure 12: Co-occurrence networks of microbial genera (pink) and host gene expression (orange = clock, green = immune, blue = corticotropin) in healthy (top) and *Argulus*-infected (bottom) trout under 24:0 LD. Node and label size scaled to degree centrality score. Label colour denotes rhythmicity (black = rhythm FDR p-value <0.05, grey = rhythm FDR p-value >0.05). Connection colour indicates association (grey = positive, red = negative, determined by Spearman correlation tests) and connection width scaled to correlation strength (thicker lines denote a higher correlation coefficient).
