# Supplementary material for: Circadian dynamics of the teleost skin immune-microbiome interface
Source: Microbiome. 2021 Nov 16;9:222. doi: 10.1186/s40168-021-01160-4 (PMC8594171; doi:10.1186/s40168-021-01160-4)
Supplement: Supplementary file 12 — Additional file 11. Supplementary Datafile 1 [file 40168_2021_1160_MOESM12_ESM.pdf]

ID	Desc
POLYAMINSYN3-PWY	polyamine biosynthesis II
PWY-2942	L-lysine biosynthesis III
PWY-4361	S-methyl-5-thio-α-D-ribose 1-phosphate degradation I
PWY-7527	L-methionine salvage cycle III
PWY-5097	L-lysine biosynthesis VI
PWY-6165	chorismate biosynthesis II (archaea)
PWY0-1241	ADP-L-glycero-β-D-manno-heptose biosynthesis
P241-PWY	coenzyme B biosynthesis
PWY-6654	phosphopantothenate biosynthesis III (archaebacteria)
PWY-5920	heme b biosynthesis from glycine
NAD-BIOSYNTHESIS-II	NAD salvage pathway III (to nicotinamide riboside)
PWY-7221	guanosine ribonucleotides de novo biosynthesis
PWY-7228	guanosine nucleotides de novo biosynthesis I
PWY-6609	adenine and adenosine salvage III
PWY-6125	guanosine nucleotides de novo biosynthesis II
PWY-6174	mevalonate pathway II (haloarchaea)
PWY-7391	isoprene biosynthesis II
PWY-7431	aromatic biogenic amine degradation (bacteria)
PWY-5022	4-aminobutanoate degradation V
PWY-5181	toluene degradation III (aerobic) (via p-cresol)
CATECHOL-ORTHO-CLEAVAGE-PWY	catechol degradation to β-ketoadipate
PWY-5417	catechol degradation III (ortho-cleavage pathway)
PWY-5431	aromatic compounds degradation via β-ketoadipate
PWY-5183	aerobic toluene degradation
CODH-PWY	reductive acetyl coenzyme A pathway I (homoacetogenic
PWY-6572	chondroitin sulfate degradation I (bacterial)
PWY-3801	sucrose degradation II (sucrose synthase)
PWY-6737	starch degradation V
PWY-6944	androstenedione degradation
LIPASYN-PWY	phospholipases
PWY0-1296	purine ribonucleosides degradation
PWY0-1297	purine deoxyribonucleosides degradation
PWY-6353	purine nucleotides degradation II (aerobic)
P221-PWY	octane oxidation
PWY-7237	myo-, chiro- and scyllo-inositol degradation
PWY0-1338	*polymyxin resistance*
GLYCOLYSIS	glycolysis I (from glucose 6-phosphate)
CALVIN-PWY	Calvin-Benson-Bassham cycle
P105-PWY	TCA cycle IV (2-oxoglutarate decarboxylase)
PWY-6969	TCA cycle V (2-oxoglutarate:ferredoxin oxidoreductase)

eJTK_BF	Peak	AvProp	Superclass
0.01458275	12.88595	0.003084	Biosynthesis
0.00510748	2.627986	0.00547015	Biosynthesis
0.01252049	6.427066	1.66E-05	Biosynthesis
0.0127468	6.325732	2.57E-05	Biosynthesis
0.02253547	2.782936	0.00530019	Biosynthesis
0.003653	2.972316	2.38E-05	Biosynthesis
0.04602597	14.17549	0.00266054	Biosynthesis
0.00022438	23.21666	1.87E-06	Biosynthesis
0.00321305	2.965556	1.78E-05	Biosynthesis
0.02084569	0.2532651	0.00113167	Biosynthesis
0.02931922	21.53345	0.00166294	Biosynthesis
0.02150775	0.2965713	0.00480853	Biosynthesis
0.03818027	0.9234364	0.0052048	Biosynthesis
0.04602597	1.897756	0.00132033	Biosynthesis
0.04939549	0.837666	0.00508615	Biosynthesis
0.0016419	1.666899	1.13E-05	Biosynthesis
0.0337347	2.790154	2.04E-05	Biosynthesis
0.01711526	15.31357	0.0024523	Degradation/Utilization/Assimilation
0.04124837	13.75506	0.00400379	Degradation/Utilization/Assimilation
0.02698494	15.82952	0.00345912	Degradation/Utilization/Assimilation
0.0164469	14.8543475	0.00274011	Degradation/Utilization/Assimilation
0.01711526	15.0864067	0.00272625	Degradation/Utilization/Assimilation
0.01711526	15.0863985	0.00272625	Degradation/Utilization/Assimilation
0.02684517	21.53345	0.00012944	Degradation/Utilization/Assimilation
0.03977813	21.01987	9.81E-06	Degradation/Utilization/Assimilation
0.0013237	5.314672	2.31E-06	Degradation/Utilization/Assimilation
0.00358437	2.359213	1.16E-05	Degradation/Utilization/Assimilation
0.03427187	13.9716708	0.00441379	Degradation/Utilization/Assimilation
0.0259928	21.85183	8.86E-06	Degradation/Utilization/Assimilation
0.04823662	5.314672	1.17E-05	Degradation/Utilization/Assimilation
0.03427187	1.388168	0.0008885	Degradation/Utilization/Assimilation
0.03975825	1.931602	0.0010104	Degradation/Utilization/Assimilation
0.04124837	1.619075	0.00216428	Degradation/Utilization/Assimilation
0.03301205	15.7577179	0.00372876	Degradation/Utilization/Assimilation
0.02948037	16.4732668	0.0050147	Degradation/Utilization/Assimilation
0.02167555	*14.88154*	*0.00235998*	*Detoxification*
0.02530526	1.35234	0.00421532	Generation of Precursor Metabolites [illegible]
0.01868302	0.5373235	0.00504978	Generation of Precursor Metabolites [illegible]
0.00488907	1.918829	0.00485251	Generation of Precursor Metabolites [illegible]
0.00634214	1.991988	0.00486768	Generation of Precursor Metabolites [illegible]

**Subclass**
Amine and Polyamine Biosynthesis
Amino Acid Biosynthesis
Amino Acid Biosynthesis
Amino Acid Biosynthesis
Amino Acid Biosynthesis
Aromatic Compound Biosynthesis
Carbohydrate Biosynthesis
Cofactor, Carrier, and Vitamin Biosynthesis
Cofactor, Carrier, and Vitamin Biosynthesis
Cofactor, Carrier, and Vitamin Biosynthesis
Cofactor, Carrier, and Vitamin Biosynthesis
Nucleoside and Nucleotide Biosynthesis
Nucleoside and Nucleotide Biosynthesis
Nucleoside and Nucleotide Biosynthesis
Nucleoside and Nucleotide Biosynthesis
Secondary Metabolite Biosynthesis
Secondary Metabolite Biosynthesis
Amine and Polyamine Degradation
Amine and Polyamine Degradation
Amino Acid Degradation
Aromatic Compound Degradation
Aromatic Compound Degradation
Aromatic Compound Degradation
Aromatic Compound Degradation
C1 Compound Utilization and Assimilation
Carbohydrate Degradation
Carbohydrate Degradation
Carbohydrate Degradation
Fatty Acid and Lipid Degradation
Fatty Acid and Lipid Degradation
Nucleoside and Nucleotide Degradation
Nucleoside and Nucleotide Degradation
Nucleoside and Nucleotide Degradation
Other
Secondary Metabolite Degradation
Antibiotic Resistance
Glycolysis
Photosynthesis
TCA Cycle
TCA Cycle

ID	Desc
PWY-5189	tetrapyrrole biosynthesis II (from glycine)
PWY-6897	thiamine diphosphate salvage II
COA-PWY	coenzyme A biosynthesis I (prokaryotic)
PWY-5188	tetrapyrrole biosynthesis I (from glutamate)
PWY-841	purine nucleotides de novo biosynthesis I
PWY-7234	inosine-5'-phosphate biosynthesis III
PWY-6123	inosine-5'-phosphate biosynthesis I
PWY-5103	L-isoleucine biosynthesis III
POLYISOPRENSYN-PWY	polyisoprenoid biosynthesis (E. coli)
PWY-4361	S-methyl-5-thio-α-D-ribose 1-phosphate de
PWY-7527	L-methionine salvage cycle III
PWY-6126	adenosine nucleotides de novo biosynthesi
PWY-5686	UMP biosynthesis I
PWY-7229	adenosine nucleotides de novo biosynthesi
HISTSYN-PWY	L-histidine biosynthesis
PWY-7219	adenosine ribonucleotides de novo biosynt
P261-PWY	coenzyme M biosynthesis I
PWY0-162	pyrimidine ribonucleotides de novo biosynth
PWY0-1479	tRNA processing
BRANCHED-CHAIN-AA-SYN-PWY	branched chain amino acid biosynthesis
PWY-5918	heme b biosynthesis from glutamate
ILEUSYN-PWY	L-isoleucine biosynthesis I (from threonine)
VALSYN-PWY	L-valine biosynthesis
PWY-5101	L-isoleucine biosynthesis II
PWY-6174	mevalonate pathway II (haloarchaea)
PWY-3781	aerobic respiration I (cytochrome c)
HEMESYN2-PWY	heme b biosynthesis II (oxygen-independen
PWY-5183	aerobic toluene degradation
PWY0-845	pyridoxal 5'-phosphate biosynthesis and sa
PWY-7391	*isoprene biosynthesis II*
GLUCONEO-PWY	gluconeogenesis I
PWY-6700	queuosine biosynthesis I (de novo)
PWY-5913	partial TCA cycle (obligate autotrophs)
TRNA-CHARGING-PWY	tRNA charging
PEPTIDOGLYCANSYN-PWY	peptidoglycan biosynthesis I (meso-diamin
TRPSYN-PWY	L-tryptophan biosynthesis
PWY-3001	L-isoleucine biosynthesis I
HEME-BIOSYNTHESIS-II	heme b biosynthesis I (aerobic)
PWY-6125	guanosine nucleotides de novo biosynthesi
PWY-1882	C1 compounds oxidation to CO2
PWY-2942	L-lysine biosynthesis III
PWY-7220	adenosine deoxyribonucleotides de novo b
PWY-7222	guanosine deoxyribonucleotides de novo b
PWY-7221	guanosine ribonucleotides de novo biosynt
HSERMETANA-PWY	L-methionine biosynthesis III
PWY-5097	L-lysine biosynthesis VI
PWY-7228	guanosine nucleotides de novo biosynthesi
PWY-6387	UDP-N-acetylmuramoyl-pentapeptide biosy
PWY-5531	*3,8-divinyl-chlorophyllide a biosynthesis II (*
PWY-7159	
PWY-5198	factor 420 biosynthesis II (mycobacteria)
PWY-7208	pyrimidine nucleobases salvage

PWY-6969	TCA cycle V (2-oxoglutarate:ferredoxin oxi
CALVIN-PWY	Calvin-Benson-Bassham cycle
PWY-5022	4-aminobutanoate degradation V
PWY0-1241	ADP-L-glycero-β-D-manno-heptose biosyn
PWY0-1061	L-alanine biosynthesis
P105-PWY	TCA cycle IV (2-oxoglutarate decarboxylas
PWY-6386	UDP-N-acetylmuramoyl-pentapeptide biosy
GLYCOLYSIS	glycolysis I (from glucose 6-phosphate)
PWY0-1338	polymyxin resistance
PWY-6385	peptidoglycan biosynthesis III (mycobacteri
UDPNAGSYN-PWY	*UDP-N-acetyl-D-glucosamine biosynthesis*
PWY-5505	L-glutamate and L-glutamine biosynthesis
PWY-7237	myo-, chiro- and scyllo-inositol degradation
PWY-6944	
PWY-6165	
NAGLIPASYN-PWY	
PWY-6654	
CATECHOL-ORTHO-CLEAVAGE-PWY	catechol degradation to β-ketoadipate
PWY-7094	fatty acid salvage
POLYAMINSYN3-PWY	polyamine biosynthesis II
CHLOROPHYLL-SYN	
P184-PWY	protocatechuate degradation I (meta-cleav
PANTOSYN-PWY	coenzyme A biosynthesis I (bacteria)
P562-PWY	myo-inositol degradation I
PWY-5417	catechol degradation III (ortho-cleavage pa
PWY-5431	aromatic compounds degradation via β-ket
PWY-7211	pyrimidine deoxyribonucleotides de novo b
PENTOSE-P-PWY	pentose phosphate pathway
PWY0-1533	methylphosphonate degradation I
PWY-7431	aromatic biogenic amine degradation (bact
PWY-7197	pyrimidine deoxyribonucleotide phosphoryl
PWY-7376	cob(II)yrinate a,c-diamide biosynthesis II (l
PROTOCATECHUATE-ORTHO-CLEAVAGE-PWY	protocatechuate degradation II (ortho-clea
HISDEG-PWY	L-histidine degradation I
PWY-5005	biotin biosynthesis II
PWY-5747	2-methylcitrate cycle II
PWY-6151	S-adenosyl-L-methionine cycle I
PWY-6339	
PWY-6121	5-aminoimidazole ribonucleotide biosynthe
FAO-PWY	fatty acid β-oxidation I (generic)
COLANSYN-PWY	colanic acid building blocks biosynthesis
PWY-6122	5-aminoimidazole ribonucleotide biosynthe
PWY-6277	5-aminoimidazole ribonucleotide biosynthe
AST-PWY	L-arginine degradation II (AST pathway)
PWY-5028	L-histidine degradation II
PWY-5941	
PWY-6572	
P124-PWY	
PWY-5484	glycolysis II (from fructose 6-phosphate)
PPGPPMET-PWY	ppGpp metabolism
PWY-7295	L-arabinose degradation IV
FASYN-INITIAL-PWY	fatty acid biosynthesis initiation
GLUCOSE1PMETAB-PWY	glucose and glucose-1-phosphate degrada
NONMEVIPP-PWY	

PWY-7560	
P281-PWY	3-phenylpropanoate degradation
ASPASN-PWY	L-aspartate and L-asparagine biosynthesis
PWY-5430	meta cleavage pathway of aromatic compo
PWY-3801	
PWY-6737	starch degradation V
DENITRIFICATION-PWY	
PWY-5384	sucrose degradation IV (sucrose phosphor
GALLATE-DEGRADATION-I-PWY	gallate degradation II
PWY-5154	L-arginine biosynthesis III (via N-acetyl-L-c
PWY-7323	GDP-mannose-derived O-antigen building
METHYLGALLATE-DEGRADATION-PWY	methylgallate degradation
GLYCOLYSIS-TCA-GLYOX-BYPASS	glycolysis, pyruvate dehydrogenase, TCA,
PWY-5177	
P162-PWY	
THRESYN-PWY	L-threonine biosynthesis
GLYCOCAT-PWY	glycogen degradation I
CRNFORCAT-PWY	creatinine degradation I
PWY-5104	L-isoleucine biosynthesis IV
PWY-6628	L-phenylalanine biosynthesis
PWY-5304	
KETOGLUCONMET-PWY	ketogluconate metabolism
ANAEROFRUCAT-PWY	homolactic fermentation
PWY-6630	L-tyrosine biosynthesis
PWY-7374	
PWY-621	sucrose degradation III (sucrose invertase)
PWY-6467	Kdo transfer to lipid IVA III (Chlamydia)
PWY-5181	toluene degradation III (aerobic) (via p-cres
P621-PWY	
1CMET2-PWY	folate transformations III (E. coli)
TYRFUMCAT-PWY	L-tyrosine degradation I
PWYG-321	mycolate biosynthesis
PWY-6470	

eJTK_BF	Peak	AvProp	Superclass	Subclass
1.9769E-07	21.54656	0.0048215	Biosynthesis	Tetrapyrrole Biosynthesis
6.08E-07	21.39857	0.00475505	Biosynthesis	Cofactor, Carrier, and Vitamin Biosynthesis
1.6956E-06	20.78448	0.00474703	Biosynthesis	Cofactor, Carrier, and Vitamin Biosynthesis
3.233E-06	21.16595	0.00491236	Biosynthesis	Tetrapyrrole Biosynthesis
3.6299E-06	20.70408	0.00538561	Biosynthesis	Nucleoside and Nucleotide Biosynthesis
3.8455E-06	20.48015	0.00467968	Biosynthesis	Nucleoside and Nucleotide Biosynthesis
5.1229E-06	20.68864	0.00469437	Biosynthesis	Nucleoside and Nucleotide Biosynthesis
6.0763E-06	21.6987	0.00605258	Biosynthesis	Amino Acid Biosynthesis
6.4304E-06	20.47147	0.00465169	Biosynthesis	Polyprenyl Biosynthesis
7.6484E-06	4.48257	1.40E-05	Biosynthesis	Amino Acid Biosynthesis
7.6484E-06	4.233041	1.60E-05	Biosynthesis	Amino Acid Biosynthesis
1.065E-05	20.75974	0.00575682	Biosynthesis	Nucleoside and Nucleotide Biosynthesis
1.2574E-05	20.70333	0.00557962	Biosynthesis	Nucleoside and Nucleotide Biosynthesis
1.4037E-05	20.7344	0.00599543	Biosynthesis	Nucleoside and Nucleotide Biosynthesis
1.6544E-05	20.41131	0.0051802	Biosynthesis	Amino Acid Biosynthesis
2.1702E-05	20.3798	0.00581327	Biosynthesis	Nucleoside and Nucleotide Biosynthesis
3.6354E-05	0.03907041	6.67E-06	Biosynthesis	Cofactor, Carrier, and Vitamin Biosynthesis
3.9023E-05	20.48819	0.00555099	Biosynthesis	Nucleoside and Nucleotide Biosynthesis
5.6235E-05	23.84892	0.00359766	Macromolecul	Nucleic Acid Processing
0.0001264	22.28346	0.00632471	Biosynthesis	Amino Acid Biosynthesis
0.00018886	20.73949	0.0047148	Biosynthesis	Cofactor, Carrier, and Vitamin Biosynthesis
0.00021681	20.73949	0.00683059	Biosynthesis	Amino Acid Biosynthesis
0.00021681	22.63277	0.00683059	Biosynthesis	Amino Acid Biosynthesis
0.00022755	23.14395	0.00742455	Biosynthesis	Amino Acid Biosynthesis
0.00024244	0.5374792	6.62E-06	Biosynthesis	Secondary Metabolite Biosynthesis
0.00025057	22.9034161	0.01302978	Generation of	Respiration
0.00032162	20.18175	0.00525593	Biosynthesis	Cofactor, Carrier, and Vitamin Biosynthesis
0.00037373	19.66225	0.00015046	Degradation/U	Aromatic Compound Degradation
0.00053273	11.2850558	0.00330237	Biosynthesis	Cofactor, Carrier, and Vitamin Biosynthesis
0.00058149		*2.29E-05*		
0.00068025	21.45754	0.0053221	Biosynthesis	Carbohydrate Biosynthesis
0.00074546	20.61366	0.00457884	Macromolecul	Nucleic Acid Processing
0.00078023	22.65912	0.00498716	Generation of	TCA cycle
0.00081654	20.17477	0.00506404	Biosynthesis	Aminoacyl-tRNA Charging
0.00085443	20.14463	0.00480638	Biosynthesis	Cell Structure Biosynthesis
0.00085443	19.76157	0.00532468	Biosynthesis	Amino Acid Biosynthesis
0.00089397	21.73842	0.0056171	Biosynthesis	Amino Acid Biosynthesis
0.00093524	20.16514	0.00454139	Biosynthesis	Cofactor, Carrier, and Vitamin Biosynthesis
0.00093524	21.49091	0.00520434	Biosynthesis	Nucleic Acid Processing
0.00110765	19.61249	1.26E-05	Degradation/U	C1 Compound Utilization and Assimilation
0.00114947	22.69257	0.00557642	Biosynthesis	Amino Acid Biosynthesis
0.00116991	21.22301	0.00569836	Biosynthesis	Nucleoside and Nucleotide Biosynthesis
0.00116991	21.22301	0.00569836	Biosynthesis	Nucleoside and Nucleotide Biosynthesis
0.00133624	21.64033	0.00489245	Biosynthesis	Nucleoside and Nucleotide Biosynthesis
0.00152462	21.46389	0.00500977	Biosynthesis	Amino Acid Biosynthesis
0.00170401	22.45109	0.00541741	Biosynthesis	Amino Acid Biosynthesis
0.00189503	21.62173	0.00531178	Biosynthesis	Nucleoside and Nucleotide Biosynthesis
0.00192183	19.93268	0.00484681	Biosynthesis	Cell Structure Biosynthesis
0.00225795		*5.74E-05*	*Biosynthesis*	*Tetrapyrrole Biosynthesis*
0.00225795				
0.0024496	22.63423	1.18E-05	Biosynthesis	Cofactor, Carrier, and Vitamin Biosynthesis
0.00267953	20.07163	0.00598838	Biosynthesis	Nucleoside and Nucleotide Biosynthesis

0.00321711	23.43107	0.00498248	Generation of	TCA cycle
0.00329081	21.63897	0.00516146	Generation of	Photosynthesis
0.00349478	12.0174	0.00392304	Degradation/U	Amine and Polyamine Degradation
0.00349478	11.20282	0.00260981	Biosynthesis	Carbohydrate Biosynthesis
0.00370984	19.0735	0.00524028	Biosynthesis	Amino Acid Biosynthesis
0.00379469	23.45178	0.00494792	Generation of	TCA cycle
0.00418368	20.29138	0.00467053	Biosynthesis	Cell Structure Biosynthesis
0.00428981	0.65574879	0.00422768	Generation of	Glycolysis
0.00446779	11.4186	0.00223592	Detoxification	Antibiotic Resistance
0.0047134	20.21351	0.00469892	Biosynthesis	Cell Structure Biosynthesis
0.00476722		*0.00473299*	*Biosynthesis*	*Carbohydrate Biosynthesis*
0.00525071	0.34125991	0.00202591	Biosynthesis	Amino Acid Biosynthesis
0.00546545	11.2080329	0.0045965	Degradation/U	Secondary Metabolite Degradation
0.00548263				
0.00624416				
0.00657558				
0.00659878				
0.00666687	11.3398854	0.00255882	Degradation/U	Aromatic Compound Degradation
0.00666687	1.677602	0.00838107	Biosynthesis	Fatty Acid and Lipid Biosynthesis
0.00693479	10.90911	0.00296217	Biosynthesis	Amine and Polyamine Biosynthesis
0.00771064				
0.00880855	23.89241	0.00021728	Degradation/U	Aromatic Compound Degradation
0.0093592	19.78353	0.00488439	Biosynthesis	Cofactor, Carrier, and Vitamin Biosynthesis
0.00946556	11.2704824	0.00273328	Degradation/U	Secondary Metabolite Degradation
0.00946556	11.1341382	0.00252207	Degradation/U	Aromatic Compound Degradation
0.00946556	11.1341272	0.00252207	Degradation/U	Aromatic Compound Degradation
0.01017709	19.28465	0.00552088	Biosynthesis	Nucleoside and Nucleotide Biosynthesis
0.01021955	10.90585	0.00491186	Generation of	Pentose Phosphate Pathways
0.01021955	11.2089401	0.00288115	Degradation/U	Inorganic Nutrient Metabolism
0.01061698	11.4923	0.00245675	Degradation/U	Amine and Polyamine Degradation
0.01096514	19.41834	0.00460367	Biosynthesis	Nucleoside and Nucleotide Biosynthesis
0.0114549	11.3869723	0.00279599	Biosynthesis	Cofactor, Carrier, and Vitamin Biosynthesis
0.01189632	10.9019595	0.00577375	Degradation/U	Aromatic Compound Degradation
0.01235336	10.3625814	0.00425075	Degradation/U	Amino Acid Degradation
0.01280344	2.08838	1.36E-05	Biosynthesis	Cofactor, Carrier, and Vitamin Biosynthesis
0.01282651	13.56752	0.00373373	Degradation/U	Carboxylate Degradation
0.01282651	0.42860774	0.00247923	Biosynthesis	Amino Acid Biosynthesis
0.01285804				
0.01367906	18.82623	0.00562554	Biosynthesis	Nucleoside and Nucleotide Biosynthesis
0.01393654	1.313601	0.00807021	Degradation/U	Fatty Acid and Lipid Degradation
0.01422752	10.8309071	0.00469205	Biosynthesis	Carbohydrate Biosynthesis
0.01525607	19.0118	0.00517144	Biosynthesis	Nucleoside and Nucleotide Biosynthesis
0.01525607	19.01174	0.00517144	Biosynthesis	Nucleoside and Nucleotide Biosynthesis
0.01603309	11.77824	0.00301227	Degradation/U	Amino Acid Degradation
0.01663401	10.4314454	0.00370562	Degradation/U	Amino Acid Degradation
0.01684358				
0.01746672				
0.01777494				
0.01789822	0.4452275	0.0036826	Generation of	Glycolysis
0.0182592	14.18736	0.00412128	Biosynthesis	Metabolic Regulator Biosynthesis
0.01848593	23.07375	0.0001518	Degradation/U	Carbohydrate Degradation
0.01924983	11.86353	0.00805644	Biosynthesis	Fatty Acid and Lipid Biosynthesis
0.02069418	10.8865373	0.00465811	Degradation/U	Carbohydrate Degradation
0.02104114				

0.02104114				
0.02130361	2.843223	0.0008236	Degradation/U	Aromatic Compound Degradation
0.02145288	10.55693	0.00313983	Biosynthesis	Amino Acid Biosynthesis
0.02190184	20.76845	0.00013149	Degradation/U	Aromatic Compound Degradation
0.02267801				
0.02304697	10.6860237	0.00415224	Degradation/U	Carbohydrate Degradation
0.02376505				
0.02388387	10.5101434	0.00398253	Degradation/U	Carbohydrate Degradation
0.02469984	0.1431483	0.00026736	Degradation/U	Aromatic Compound Degradation
0.02474838	12.67755	0.00530625	Biosynthesis	Amino Acid Biosynthesis
0.02552565	10.4768417	0.00449515	Biosynthesis	Carbohydrate Biosynthesis
0.02559135	0.184094	0.00031995	Degradation/U	Aromatic Compound Degradation
0.02751568	0.2374286	0.00457682	Generation of	Glycolysis
0.02866037				
0.02929334				
0.0294077	21.52065	0.00513648	Biosynthesis	Amino Acid Biosynthesis
0.03275885	10.75643	0.00383843	Degradation/U	Carbohydrate Degradation
0.03391035	14.33289	0.00031199	Degradation/U	Amine and Polyamine Degradation
0.03391035	1.33327013	0.00242679	Biosynthesis	Amino Acid Biosynthesis
0.0351669	12.35229	0.00612333	Biosynthesis	Amino Acid Biosynthesis
0.03628591				
0.03694973	4.356073	0.00103866	Degradation/U	Carboxylate Degradation
0.04023668	1.42100487	0.00338385	Generation of	Fermentation
0.04023668	0.6938823	0.0048852	Biosynthesis	Amino Acid Biosynthesis
0.04105068				
0.04162311	10.2521586	0.00383702	Degradation/U	Carbohydrate Degradation
0.04300116	19.25475	0.00439794	Biosynthesis	Cell Structure Biosynthesis
0.04448228	8.66485	0.00300959	Degradation/U	Aromatic Compound Degradation
0.0456403				
0.04604482	12.2390022	0.00614593	Biosynthesis	Cofactor, Carrier, and Vitamin Biosynthesis
0.04758322	10.41203	0.00457516	Degradation/U	Amino Acid Degradation
0.04915475	13.06886	0.00834762	Biosynthesis	Fatty Acid and Lipid Biosynthesis
0.04921667				

ID	Desc	eJTK_BF	Peak
POLYAMINSYN3-PWY	polyamine biosynthesis II	0.01181769	14.24225
PWY-2942	L-lysine biosynthesis III	0.00107423	0.4640838
PWY-6151	S-adenosyl-L-methionine cycle I	0.00291762	2.50907
PWY-4361	S-methyl-5-thio-α-D-ribose 1-phc	0.00521667	1.359399
PWY-7527	L-methionine salvage cycle III	0.00521667	1.368213
PWY-5505	L-glutamate and L-glutamine bio	0.00967176	2.25966707
BRANCHED-CHAIN-AA-SYN-PWY	branched chain amino acid biosy	0.01139516	23.77327
ASPASN-PWY	L-aspartate and L-asparagine bic	0.01319197	14.90918
ILEUSYN-PWY	L-isoleucine biosynthesis I (from	0.01701832	0.2098248
VALSYN-PWY	L-valine biosynthesis	0.01701832	0.2098239
PWY-3001	L-isoleucine biosynthesis I	0.0174237	22.11811
PWY-5101	L-isoleucine biosynthesis II	0.01857189	0.6445518
PWY-6630	L-tyrosine biosynthesis	0.02842071	2.593421
PWY-5097	L-lysine biosynthesis VI	0.02930627	23.14649
GLUTORN-PWY	L-ornithine biosynthesis I	0.03610503	14.6357
PWY-5154	L-arginine biosynthesis III (via N-	0.04233608	14.9656
PWY-6165	chorismate biosynthesis II (archa	0.00010365	1.616014
PWY0-1241	ADP-L-glycero-β-D-manno-hepto	0.02964945	14.7731551
PWY-6749	CMP-legionaminate biosynthesis	0.03013338	0.2248954
PWY-5659	GDP-mannose biosynthesis	0.03610503	15.2239192
PWY-6167	flavin biosynthesis II (archaea)	6.5205E-05	1.643688
PWY-6654	phosphopantothenate biosynthes	0.00010365	1.637631
PWY0-845	pyridoxal 5'-phosphate biosynthe	0.00141469	14.1404798
P261-PWY	coenzyme M biosynthesis I	0.007646	23.25483
BIOTIN-BIOSYNTHESIS-PWY	biotin biosynthesis I	0.01011498	14.26564
1CMET2-PWY	folate transformations III (E. coli)	0.02301099	15.01738
PWY-5198	factor 420 biosynthesis II (mycob	0.02949096	2.445492
PWY-5920	heme b biosynthesis from glycine	0.02964945	1.46966324
PWY-7376	cob(II)yrinate a,c-diamide biosyn	0.03071576	14.9517
PYRIDOXSYN-PWY	pyridoxal 5'-phosphate biosynthe	0.03071576	14.20977
FOLSYN-PWY	tetrahydrofolate biosynthesis anc	0.04069345	15.34843
PWY-7663	gondoate biosynthesis (anaerobi	0.00439425	14.68228
PWYG-321	mycolate biosynthesis	0.00587285	14.54942
PWY-7664	oleate biosynthesis IV (anaerobic	0.01129236	14.75275
FASYN-ELONG-PWY	fatty acid elongation -- saturated	0.01181769	14.63671
PWY0-862	(5Z)-dodecenoate biosynthesis I	0.01353166	15.04154
FASYN-INITIAL-PWY	fatty acid biosynthesis initiation (I	0.01927897	14.4426076
PWY-6282	palmitoleate biosynthesis I (from	0.0249699	14.64343
PPGPPMET-PWY	ppGpp metabolism	0.00703722	14.32313
PWY-7221	guanosine ribonucleotides de no	0.00322773	23.1981
PWY-6125	guanosine nucleotides de novo b	0.00333618	22.47322
PWY-7228	guanosine nucleotides de novo b	0.00393722	22.85595
PWY-7200	pyrimidine deoxyribonucleoside s	0.01439528	2.388056
PWY-7220	adenosine deoxyribonucleotides	0.01903241	20.60992
PWY-7222	guanosine deoxyribonucleotides	0.01903241	20.60982
PWY-7199	pyrimidine deoxyribonucleosides	0.02292211	2.079752
PWY-841	purine nucleotides de novo biosy	0.02470772	21.56337
PWY-6609	adenine and adenosine salvage	0.03610503	2.16887792
PWY-6122	5-aminoimidazole ribonucleotide	0.04623035	20.8905
PWY-6277	5-aminoimidazole ribonucleotide	0.04623035	20.89054
PWY-6519	8-amino-7-oxononanoate biosyn	0.04762899	14.0817419
POLYISOPRENSYN-PWY	polyisoprenoid biosynthesis (E. c	0.01524305	19.21801

PWY-6581	spirilloxanthin and 2,2'-diketo-spi	0.00198822	23.27743
PWY-6174	mevalonate pathway II (haloarch	0.00479574	23.62934
ENTBACSYN-PWY	enterobactin biosynthesis	0.04486645	2.531403
CRNFORCAT-PWY	creatinine degradation I	0.00299958	14.82222
PWY-5022	4-aminobutanoate degradation V	0.00703722	14.20711
PWY-7431	aromatic biogenic amine degrada	0.03198989	14.72142
PWY-6505	L-tryptophan degradation XII (Ge	0.0101952	2.545333
AST-PWY	L-arginine degradation II (AST pa	0.02103614	14.3173977
PWY-5655	L-tryptophan degradation IX	0.03604063	2.545476
PWY-6210	2-aminophenol degradation	0.01037128	2.455527
CATECHOL-ORTHO-CLEAVAGE-PWY	catechol degradation to β-ketoac	0.01364088	14.15661
PWY-5431	aromatic compounds degradatio	0.02835517	14.27361
PWY-5430	meta cleavage pathway of aroma	0.02909483	3.131856
PWY-5417	catechol degradation III (ortho-cl	0.02835517	14.273605
PWY-5647	2-nitrobenzoate degradation I	0.03604063	2.28502
PWY-1882	C1 compounds oxidation to CO2	0.03461024	21.64286
DHGLUCONATE-PYR-CAT-PWY	glucose degradation (oxidative)	0.00246111	12.14957
PWY-5747	2-methylcitrate cycle II	0.02716486	13.92641
PWY-5654	2-amino-3-carboxymuconate ser	0.03604063	2.199813
PWY-6641	sulfolactate degradation	0.00251725	1.530559
SO4ASSIM-PWY	assimilatory sulfate reduction I	0.02301099	14.2719838
PWY0-1533	methylphosphonate degradation	0.02964945	14.54508
PWY0-1297	purine deoxyribonucleosides deg	0.01785758	2.266681
P164-PWY	purine nucleobases degradation	0.03403874	0.00405378
PWY-6353	purine nucleotides degradation II	0.03757968	2.27563982
PWY0-1296	purine ribonucleosides degradati	0.02206736	2.125236
P221-PWY	octane oxidation	0.00807222	14.3452877
PWY0-1261	anhydromuropeptides recycling I	0.02013786	2.90950758
PWY-7237	myo-, chiro- and scyllo-inositol de	0.04069345	14.8459775
PWY0-1338	polymyxin resistance	0.00844739	14.28478
ANAEROFRUCAT-PWY	homolactic fermentation	0.0210286	2.898976
GLYCOLYSIS	glycolysis I (from glucose 6-phos	0.02395495	2.693878
PWY-5484	glycolysis II (from fructose 6-pho	0.0380901	2.759216
PENTOSE-P-PWY	pentose phosphate pathway	0.00462354	15.03732
PWY-181	photorespiration	0.02964945	3.082635
CALVIN-PWY	Calvin-Benson-Bassham cycle	0.03225926	22.72633
PWY-3781	aerobic respiration I (cytochrome	0.02508665	2.69166991
P105-PWY	TCA cycle IV (2-oxoglutarate dec	0.00528626	2.622187
PWY-6969	TCA cycle V (2-oxoglutarate:ferr	0.00554865	2.535885
PWY-7254	TCA cycle VII (acetate-producers	0.00906749	13.1901661
REDCITCYC	TCA cycle VI (Helicobacter)	0.00967176	13.43173
TCA-GLYOX-BYPASS	glyoxylate bypass and TCA	0.03198989	3.504293

AvProp	Superclass	Subclass
0.00298841	Biosynthesis	Amine and Polyamine Biosynthesis
0.00550471	Biosynthesis	Amino Acid Biosynthesis
0.00281659	Biosynthesis	Amino Acid Biosynthesis
1.0959E-06	Biosynthesis	Amino Acid Biosynthesis
1.8009E-06	Biosynthesis	Amino Acid Biosynthesis
0.00195807	Biosynthesis	Amino Acid Biosynthesis
0.00626044	Biosynthesis	Amino Acid Biosynthesis
0.00319298	Biosynthesis	Amino Acid Biosynthesis
0.00683603	Biosynthesis	Amino Acid Biosynthesis
0.00683603	Biosynthesis	Amino Acid Biosynthesis
0.00549441	Biosynthesis	Amino Acid Biosynthesis
0.0074879	Biosynthesis	Amino Acid Biosynthesis
0.0050413	Biosynthesis	Amino Acid Biosynthesis
0.00531615	Biosynthesis	Amino Acid Biosynthesis
0.00601301	Biosynthesis	Amino Acid Biosynthesis
0.00520577	Biosynthesis	Amino Acid Biosynthesis
5.089E-06	Biosynthesis	Aromatic Compound Biosynthesis
0.00260498	Biosynthesis	Carbohydrate Biosynthesis
2.3537E-05	Biosynthesis	Carbohydrate Biosynthesis
0.00587121	Biosynthesis	Carbohydrate Biosynthesis
6.3302E-06	Biosynthesis	Cofactor, Carrier, and Vitamin Biosynthesis
3.8116E-06	Biosynthesis	Cofactor, Carrier, and Vitamin Biosynthesis
0.00343884	Biosynthesis	Cofactor, Carrier, and Vitamin Biosynthesis
2.6094E-06	Biosynthesis	Cofactor, Carrier, and Vitamin Biosynthesis
0.00508217	Biosynthesis	Cofactor, Carrier, and Vitamin Biosynthesis
0.00597742	Biosynthesis	Cofactor, Carrier, and Vitamin Biosynthesis
9.1684E-06	Biosynthesis	Cofactor, Carrier, and Vitamin Biosynthesis
0.001248	Biosynthesis	Cofactor, Carrier, and Vitamin Biosynthesis
0.00289434	Biosynthesis	Cofactor, Carrier, and Vitamin Biosynthesis
0.00387373	Biosynthesis	Cofactor, Carrier, and Vitamin Biosynthesis
0.00555335	Biosynthesis	Cofactor, Carrier, and Vitamin Biosynthesis
0.00849158	Biosynthesis	Fatty Acid and Lipid Biosynthesis
0.00810194	Biosynthesis	Fatty Acid and Lipid Biosynthesis
0.00730762	Biosynthesis	Fatty Acid and Lipid Biosynthesis
0.00760872	Biosynthesis	Fatty Acid and Lipid Biosynthesis
0.00694986	Biosynthesis	Fatty Acid and Lipid Biosynthesis
0.00799134	Biosynthesis	Fatty Acid and Lipid Biosynthesis
0.00716247	Biosynthesis	Fatty Acid and Lipid Biosynthesis
0.00386578	Biosynthesis	Metabolic Regulator Biosynthesis
0.00483871	Biosynthesis	Nucleoside and Nucleotide Biosynthesis
0.00509612	Biosynthesis	Nucleoside and Nucleotide Biosynthesis
0.00521677	Biosynthesis	Nucleoside and Nucleotide Biosynthesis
0.00235527	Biosynthesis	Nucleoside and Nucleotide Biosynthesis
0.00551023	Biosynthesis	Nucleoside and Nucleotide Biosynthesis
0.00551023	Biosynthesis	Nucleoside and Nucleotide Biosynthesis
0.00147325	Biosynthesis	Nucleoside and Nucleotide Biosynthesis
0.0052573	Biosynthesis	Nucleoside and Nucleotide Biosynthesis
0.00136183	Biosynthesis	Nucleoside and Nucleotide Biosynthesis
0.00510372	Biosynthesis	Nucleoside and Nucleotide Biosynthesis
0.00510372	Biosynthesis	Nucleoside and Nucleotide Biosynthesis
0.00590119	Biosynthesis	Other
0.00448635	Biosynthesis	Polyprenyl Biosynthesis

2.7928E-06	Biosynthesis	Secondary Metabolite Biosynthesis
2.1503E-06	Biosynthesis	Secondary Metabolite Biosynthesis
0.00117934	Biosynthesis	Secondary Metabolite Biosynthesis
0.0003129	Degradation/U	Amine and Polyamine Degradation
0.00380292	Degradation/U	Amine and Polyamine Degradation
0.00229814	Degradation/U	Amine and Polyamine Degradation
2.7211E-05	Degradation/U	Amino Acid Degradation
0.0028984	Degradation/U	Amino Acid Degradation
6.5895E-05	Degradation/U	Amino Acid Degradation
1.1082E-05	Degradation/U	Aromatic Compound Degradation
0.00269879	Degradation/U	Aromatic Compound Degradation
0.00269496	Degradation/U	Aromatic Compound Degradation
9.4917E-05	Degradation/U	Aromatic Compound Degradation
0.00269496	Degradation/U	Aromatic Compound Degradation
3.4473E-05	Degradation/U	Aromatic Compound Degradation
2.2415E-06	Degradation/U	C1 Compound Utilization and Assimilation
0.00072437	Degradation/U	Carbohydrate Degradation
0.00351219	Degradation/U	Carboxylate Degradation
2.9498E-05	Degradation/U	Carboxylate Degradation
3.7385E-06	Degradation/U	Inorganic Nutrient Metabolism
0.00698497	Degradation/U	Inorganic Nutrient Metabolism
0.00294204	Degradation/U	Inorganic Nutrient Metabolism
0.00106358	Degradation/U	Nucleoside and Nucleotide Degradation
0.0002128	Degradation/U	Nucleoside and Nucleotide Degradation
0.00222637	Degradation/U	Nucleoside and Nucleotide Degradation
0.00095736	Degradation/U	Nucleoside and Nucleotide Degradation
0.00351393	Degradation/U	Other
0.00437805	Degradation/U	Secondary Metabolite Degradation
0.00493102	Degradation/U	Secondary Metabolite Degradation
0.002264	Detoxification	Antibiotic Resistance
0.00371325	Generation of	Fermentation
0.00430438	Generation of	Glycolysis
0.00386352	Generation of	Glycolysis
0.00509678	Generation of	Pentose Phosphate Pathways
0.00260144	Generation of	Photosynthesis
0.0050757	Generation of	Photosynthesis
0.01279567	Generation of	Respiration
0.004922	Generation of	TCA cycle
0.00495477	Generation of	TCA cycle
0.00610661	Generation of	TCA cycle
0.00637222	Generation of	TCA cycle
0.00592074	Generation of	TCA cycle

ID	Desc	eJTK_BF	Peak
PWY-5505	L-glutamate and L-g	0.001421708	3.970232131
ILEUSYN-PWY	L-isoleucine biosynth	0.004543789	4.345035
VALSYN-PWY	L-valine biosynthesis	0.004543789	4.345035
HSERMETANA-PWY	L-methionine biosyn	0.005581124	3.037407
BRANCHED-CHAIN-AA-SYN-PWY	branched chain amir	0.006054548	2.962367
PWY-3001	L-isoleucine biosynth	0.006143178	1.443449
PWY-5101	L-isoleucine biosynth	0.009394609	4.222059
THRESYN-PWY	L-threonine biosynth	0.009644006	0.5940647
PWY-5097	L-lysine biosynthesis	0.026371586	0.9127464
PWY-5104	L-isoleucine biosynth	0.034570952	3.902741
HISTSYN-PWY	L-histidine biosynthe	0.043492245	22.16943
ARGSYNBSUB-PWY	L-arginine biosynthe	0.045053243	18.09423
DAPLYSINESYN-PWY	L-lysine biosynthesis	0.048327538	19.53305
PWY-6165	chorismate biosynthe	0.002854316	1.615874
PWY0-1241	ADP-L-glycero-β-D-r	0.004359157	15.23928214
UDPNAGSYN-PWY	UDP-N-acetyl-D-gluc	0.022574372	20.28038
OANTIGEN-PWY	O-antigen building bl	0.031490649	19.55511
GLYCOGENSYNTH-PWY	glycogen biosynthes	0.001774644	14.70316207
PWY0-845	pyridoxal 5'-phospha	0.00274041	15.0958907
PWY-6167	flavin biosynthesis II	0.003190803	0.8689589
NADSYN-PWY	NAD de novo biosyn	0.005509694	5.05939
PYRIDNUCSAL-PWY	NAD salvage pathwa	0.008020816	17.18851
PWY-6654	phosphopantothenat	0.009416284	0.9057542
PYRIDOXSYN-PWY	pyridoxal 5'-phospha	0.012815443	16.00489
PYRIDNUCSYN-PWY	NAD de novo biosyn	0.02345085	18.96047
P381-PWY	adenosylcobalamin b	0.027474896	4.741932
NAD-BIOSYNTHESIS-II	NAD salvage pathwa	0.030275161	3.154132
PWY-7539	6-hydroxymethyl-dihy	0.037578797	17.36494
UBISYN-PWY	ubiquinol-8 biosynthe	0.046664541	19.28877
PWY-5973	cis-vaccenate biosyn	0.012255232	3.30482
PWY-5989	stearate biosynthesis	0.027365974	9.254426
PPGPPMET-PWY	ppGpp metabolism	0.001954237	17.83819
PWY-7197	pyrimidine deoxyribo	0.00351924	20.47036
PWY-7211	pyrimidine deoxyribo	0.046664541	21.14813
PWY-6519	8-amino-7-oxononar	0.0194696	17.38199359
PWY-7392	taxadiene biosynthes	0.016115597	5.105335729
NONMEVIPP-PWY	methylerythritol phos	0.020936278	20.3298
PWY-7560	methylerythritol phos	0.020936278	20.3298
PWY-5188	tetrapyrrole biosynth	0.01231821	0.5289638
PWY-5189	tetrapyrrole biosynth	0.01616326	0.7363197
PWY-5022	4-aminobutanoate de	0.026949007	16.45844026
PWY-5651	L-tryptophan degrad	0.00598376	5.136845
PWY-5088	L-glutamate degrada	0.015852445	15.48333
PWY-5655	L-tryptophan degrad	0.018159856	4.998904
THREOCAT-PWY	L-threonine metaboli	0.023239897	4.501625
AST-PWY	L-arginine degradatio	0.020887282	16.51537
GALLATE-DEGRADATION-I-PWY	gallate degradation I	0.011866639	4.077029189
METHYLGALLATE-DEGRADATION-PWY	methylgallate degrac	0.012815443	4.012823
PWY-5647	2-nitrobenzoate degr	0.021102367	5.078541
P184-PWY	protocatechuate deg	0.02331383	3.670533
P281-PWY	3-phenylpropanoate	0.025241389	6.233675
PWY-5178	toluene degradation	0.037508947	4.922143

PWY-5420	catechol degradation	0.04627134	5.325998
GLYCOCAT-PWY	glycogen degradatio	0.001554128	15.30074885
PWY-6737	starch degradation V	0.003249712	15.27246744
PWY-6901	glucose and xylose d	0.00528597	6.00861119
PWY-621	sucrose degradation	0.014925558	14.84242644
PWY-5384	sucrose degradation	0.015500719	16.05329
PWY-6713	L-rhamnose degrada	0.018593581	14.69518
GLUCOSE1PMETAB-PWY	glucose and glucose	0.033374211	15.98753504
PWY-6572	chondroitin sulfate d	0.049079073	4.743496
PWY-5654	2-amino-3-carboxym	0.022603345	5.083428
PWY-4984	urea cycle	0.018058015	4.842164
DENITRIFICATION-PWY	nitrate reduction I (d	0.018234462	3.604276
PWY490-3	nitrate reduction VI (	0.039756278	3.985901
PWY-6641	sulfolactate degrada	0.043606071	12.80359
GALACT-GLUCUROCAT-PWY	hexuronide and hexu	0.047899028	4.30078
PWY-7237	myo-, chiro- and scy	0.039756278	15.01279896
PWY0-1338	polymyxin resistance	0.022477812	15.03892
ANAEROFRUCAT-PWY	homolactic fermenta	0.005509694	4.164371
P122-PWY	heterolactic fermenta	0.026182569	4.777284
P108-PWY	pyruvate fermentatio	0.045634158	4.426327
PWY-5484	glycolysis II (from fru	0.019400183	3.671253
GLYCOLYSIS-E-D	glycolysis and the Er	0.02115146	2.70668
GLYCOLYSIS	glycolysis I (from glu	0.02166923	3.290313
ANAGLYCOLYSIS-PWY	glycolysis III (from gl	0.034570952	4.755769
PENTOSE-P-PWY	pentose phosphate p	0.048857234	15.21065
PWY-3781	aerobic respiration I	0.020131187	1.778433965
GLYCOLYSIS-TCA-GLYOX-BYPASS	glycolysis, pyruvate d	0.003620054	3.183469
PWY-6969	TCA cycle V (2-oxog	0.031092676	2.883366
P105-PWY	TCA cycle IV (2-oxog	0.03708172	2.908569

AvProp	Superclass	Subclass
0.002246188	Biosynthesis	Amino Acid Biosynthesis
0.006881252	Biosynthesis	Amino Acid Biosynthesis
0.006881252	Biosynthesis	Amino Acid Biosynthesis
0.00510453	Biosynthesis	Amino Acid Biosynthesis
0.006341953	Biosynthesis	Amino Acid Biosynthesis
0.005689249	Biosynthesis	Amino Acid Biosynthesis
0.007470301	Biosynthesis	Amino Acid Biosynthesis
0.005253538	Biosynthesis	Amino Acid Biosynthesis
0.005496256	Biosynthesis	Amino Acid Biosynthesis
0.002913613	Biosynthesis	Amino Acid Biosynthesis
0.005140456	Biosynthesis	Amino Acid Biosynthesis
0.00530721	Biosynthesis	Amino Acid Biosynthesis
0.005604531	Biosynthesis	Amino Acid Biosynthesis
5.17277E-06	Biosynthesis	Aromatic Compound Biosynthesis
0.002440588	Biosynthesis	Carbohydrate Biosynthesis
0.00466699	Biosynthesis	Carbohydrate Biosynthesis
0.004881189	Biosynthesis	Carbohydrate Biosynthesis
0.003809261	Biosynthesis	Carbohydrate Biosynthesis
0.003157525	Biosynthesis	Cofactor, Carrier, and Vitamin Biosynthesis
7.00034E-06	Biosynthesis	Cofactor, Carrier, and Vitamin Biosynthesis
0.000921375	Biosynthesis	Cofactor, Carrier, and Vitamin Biosynthesis
0.004316655	Biosynthesis	Cofactor, Carrier, and Vitamin Biosynthesis
4.38788E-06	Biosynthesis	Cofactor, Carrier, and Vitamin Biosynthesis
0.003910828	Biosynthesis	Cofactor, Carrier, and Vitamin Biosynthesis
0.004549027	Biosynthesis	Cofactor, Carrier, and Vitamin Biosynthesis
0.000192164	Biosynthesis	Cofactor, Carrier, and Vitamin Biosynthesis
0.001814153	Biosynthesis	Cofactor, Carrier, and Vitamin Biosynthesis
0.005768482	Biosynthesis	Cofactor, Carrier, and Vitamin Biosynthesis
0.005099068	Biosynthesis	Cofactor, Carrier, and Vitamin Biosynthesis
0.00775419	Biosynthesis	Fatty Acid and Lipid Biosynthesis
0.007646438	Biosynthesis	Fatty Acid and Lipid Biosynthesis
0.003971792	Biosynthesis	Metabolic Regulator Biosynthesis
0.004539271	Biosynthesis	Nucleoside and Nucleotide Biosynthesis
0.005455668	Biosynthesis	Nucleoside and Nucleotide Biosynthesis
0.006187973	Biosynthesis	Other
0.001312291	Biosynthesis	Secondary Metabolite Biosynthesis
0.004416301	Biosynthesis	Secondary Metabolite Biosynthesis
0.004416301	Biosynthesis	Secondary Metabolite Biosynthesis
0.004890666	Biosynthesis	Tetrapyrrole Biosynthesis
0.004804664	Biosynthesis	Tetrapyrrole Biosynthesis
0.003690869	Degradation/Utilization/Assimilation	Amine and Polyamine Degradation
0.000641595	Degradation/Utilization/Assimilation	Amino Acid Degradation
3.75225E-06	Degradation/Utilization/Assimilation	Amino Acid Degradation
9.59624E-05	Degradation/Utilization/Assimilation	Amino Acid Degradation
7.16386E-05	Degradation/Utilization/Assimilation	Amino Acid Degradation
0.003072328	Degradation/Utilization/Assimilation	Amino Acid Degradation
0.000397	Degradation/Utilization/Assimilation	Aromatic Compound Degradation
0.000457403	Degradation/Utilization/Assimilation	Aromatic Compound Degradation
4.9579E-05	Degradation/Utilization/Assimilation	Aromatic Compound Degradation
0.00033991	Degradation/Utilization/Assimilation	Aromatic Compound Degradation
0.000882233	Degradation/Utilization/Assimilation	Aromatic Compound Degradation
0.000343784	Degradation/Utilization/Assimilation	Aromatic Compound Degradation

0.000423899	Degradation/Utilization/Assimilation	Aromatic Compound Degradation
0.003742478	Degradation/Utilization/Assimilation	Carbohydrate Degradation
0.00402326	Degradation/Utilization/Assimilation	Carbohydrate Degradation
0.001292317	Degradation/Utilization/Assimilation	Carbohydrate Degradation
0.003793939	Degradation/Utilization/Assimilation	Carbohydrate Degradation
0.004027744	Degradation/Utilization/Assimilation	Carbohydrate Degradation
4.62587E-06	Degradation/Utilization/Assimilation	Carbohydrate Degradation
0.004558663	Degradation/Utilization/Assimilation	Carbohydrate Degradation
8.13244E-06	Degradation/Utilization/Assimilation	Carbohydrate Degradation
4.2839E-05	Degradation/Utilization/Assimilation	Carboxylate Degradation
0.001961605	Degradation/Utilization/Assimilation	Inorganic Nutrient Metabolism
0.000313593	Degradation/Utilization/Assimilation	Inorganic Nutrient Metabolism
0.000211471	Degradation/Utilization/Assimilation	Inorganic Nutrient Metabolism
1.27388E-05	Degradation/Utilization/Assimilation	Inorganic Nutrient Metabolism
0.000578658	Degradation/Utilization/Assimilation	Other
0.003953157	Degradation/Utilization/Assimilation	Secondary Metabolite Degradation
0.00193172	Detoxification	Antibiotic Resistance
0.003468229	Generation of Precursor Metabolites	Fermentation
0.00031409	Generation of Precursor Metabolites	Fermentation
0.001328978	Generation of Precursor Metabolites	Fermentation
0.004031083	Generation of Precursor Metabolites	Glycolysis
0.004387894	Generation of Precursor Metabolites	Glycolysis
0.004453307	Generation of Precursor Metabolites	Glycolysis
0.003902824	Generation of Precursor Metabolites	Glycolysis
0.004574708	Generation of Precursor Metabolites	Pentose Phosphate Pathways
0.013302668	Generation of Precursor Metabolites	Respiration
0.0045788	Generation of Precursor Metabolites	TCA cycle
0.005272762	Generation of Precursor Metabolites	TCA cycle
0.005213865	Generation of Precursor Metabolites	TCA cycle
